# Phage-triggered reverse transcription assembles a toxic repetitive gene from a non-coding RNA

**DOI:** 10.1126/science.adq3977

**Published:** 2024-10-04

**Authors:** Max E. Wilkinson, David Li, Alex Gao, Rhiannon K. Macrae, Feng Zhang

**Affiliations:** 1 Howard Hughes Medical Institute, Massachusetts Institute of Technology, Cambridge, MA 02139, USA.; 2 Broad Institute of MIT and Harvard, Cambridge, MA 02142, USA; 3 McGovern Institute for Brain Research, Massachusetts Institute of Technology, Cambridge, MA 02139, USA.; 4 Department of Brain and Cognitive Sciences, Massachusetts Institute of Technology, Cambridge, MA 02139, USA; 5 Department of Biological Engineering, Massachusetts Institute of Technology, Cambridge, MA 02139, USA.; 6 Department of Bioengineering, Stanford University, Stanford, CA 94305, USA.; 7 Department of Biochemistry, Stanford University, Stanford, CA 94305, USA.

## Abstract

Reverse transcription has frequently been co-opted for cellular functions and in prokaryotes is associated with protection against viral infection, but the underlying mechanisms of defense are generally unknown. Here, we show that in the DRT2 defense system the reverse transcriptase binds a neighboring pseudoknotted non-coding RNA. Upon bacteriophage infection, a template region of this RNA is reverse transcribed into an array of tandem repeats that reconstitute a promoter and open reading frame, allowing expression of a toxic repetitive protein and an abortive infection response. Biochemical reconstitution of this activity and cryogenic electron microscopy provide a molecular basis for repeat synthesis. Gene synthesis from a non-coding RNA is a new mode of genetic regulation in prokaryotes.

## One-Sentence Summary

Bacteria use reverse transcriptases to synthesize toxic repetitive genes in response to viral infection.

## Main Text:

RNA-templated DNA synthesis, or reverse transcription, is a fundamental way the flow of genetic information can be reversed. Catalyzed by reverse transcriptases (RTs), this activity is commonly associated with the mobility of RNA-based mobile genetic elements like retroviruses, retrotransposons, and group II introns, but has also been co-opted several times for cellular functions (1). In eukaryotes, RT activity by telomerase maintains genome integrity (2), and an RT-domain protein forms the heart of the spliceosome, which removes introns from pre-mRNA (3). In prokaryotes, RTs are commonly associated with phage defense (4, 5). For example, RTs can mediate CRISPR spacer acquisition from RNA (6), and retron RTs synthesize specialized RNA/DNA/protein complexes that function via diverse and emerging mechanisms to restrict phage propagation (4, 7). A variety of RTs can be found in prokaryotic genomes beyond these relatively well-characterized examples (8). A prominent example is the so-called “Unknown Group” RTs – or UG systems – which are distantly related to group II intron RTs but encompass at least 42 different families with considerable sequence diversity and different domain architectures (5, 9, 10). Although most UG systems have not been functionally characterized, several have been shown to provide defense against DNA phages. Some of these are therefore called defense-associated RTs, or DRTs, and some are called Abi RTs due to their ability to mediate phage defense by abortive infection (Abi) (4, 5, 11). Some Abi RTs like AbiK and Abi-P2 were shown to perform protein-primed non-templated nucleotide polymerization, but how this activity leads to phage defense is unclear (12, 13).

We were intrigued by a particular class of UG systems, the so-called Class 2 systems, due to the frequent association of their RT genes with an adjacent non-coding RNA (ncRNA) (5). This class includes several DRTs where presence of the ncRNA is required for phage defense (4, 5). We investigated the mechanism by which the Type 2 DRT (hereafter, DRT2) provides phage defense, and in doing so revealed a unique mode of gene expression where a toxic gene is assembled from a non-coding RNA.

### DRT2 systems provide phage defense by abortive infection

**Fig. 1.**
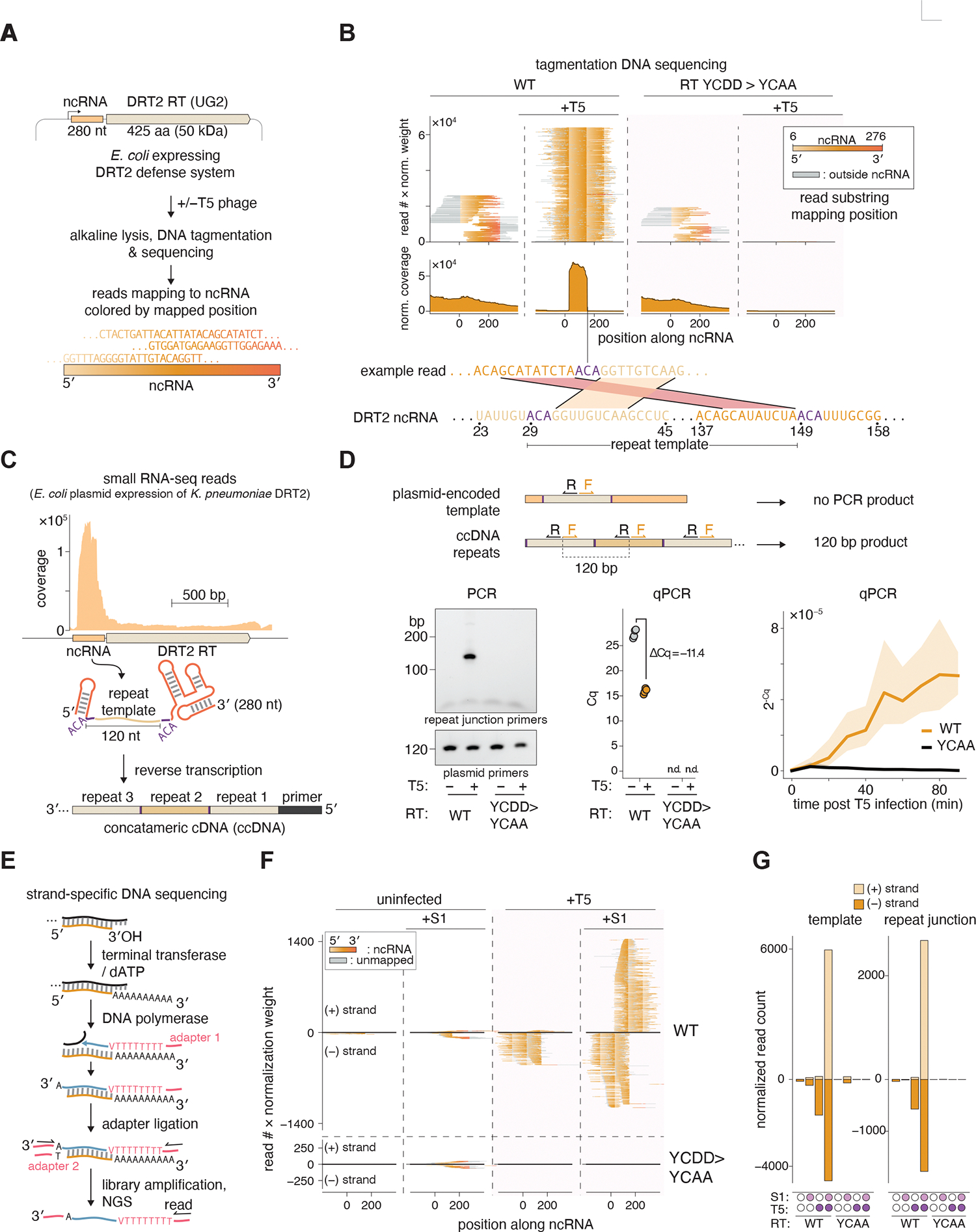
DRT2 reverse transcriptase produces concatemeric cDNA upon phage infection. **(A)** Diagram of the DRT2 defense system and schematic for assay for cDNA production by DRT2 reverse transcriptases. (**B)** Tagmentation reads were annotated by sliding an 11 bp window along their lengths and coloring according to the mapping position of the center of the window to the DRT2 ncRNA. Disjunct read coloring represents chimeric reads, an example of which is shown below the plot for the DRT2 system. Coverage is calculated using soft-clipped reads. (**C)** Small RNA sequencing reads mapped to the DRT2 locus after expression in *E. coli*. The diagram shows the proposed formation of concatemeric cDNA (ccDNA) by reverse transcription. (**D)** PCR and qPCR assay for ccDNA formation. Top: schematic for specific detection of ccDNA using outward-facing primers. Bottom left: PCR of DRT2-expressing *E. coli* with and without infection with T5 phage. YCDD>YCAA, an active site mutant (D269A and D270A) of the DRT2 reverse transcriptase. Plasmid primers are specific for a 120 bp region of the DRT2 expression plasmid backbone. Bottom middle: quantitative PCR (qPCR) of the same conditions, with two biological and two technical replicates. Bottom right: qPCR of ccDNA production during a time course of T5 infection. Shading represents the standard error of three independent replicates. (**E**) Schematic of strand-specific DNA sequencing library preparation. V indicates the DNA bases A, C, or G. (**F**) Strand-specific reads mapped to the DRT2 ncRNA, colored as in **B**, with the position above or below the x-axis corresponding to plus- or minus-strand mapping respectively. Read height is weighted by the number of reads in the same sample mapping to a single-strand DNA control. (**G**) Normalized strand-specific read counts for the reads shown in **F**. “Template” counts any read mapping within the 120 bp template region of the DRT2 ncRNA. “Repeat junction” counts any read containing juxtaposed template regions.

We had previously shown that a DRT2 system from *Klebsiella pneumoniae* provides defense against T5 phage when expressed in *Escherichia coli* (4, 5). The system consists of a 280 nt ncRNA upstream of a gene for a 425 amino acid RT-domain protein with no additional domains (Fig. 1A). By plaque assays, we validated that expression of this system in *E. coli* B strains provides 10^3^ – 10^5^-fold defense against phages T5 and ZL19 but not against T2 or T4, and that defense requires an intact RT active site and the ncRNA (fig. S1). DRT2 expression did not impact T5 adsorption to *E. coli* cells, nor did it impact cell survival after T5 infection (fig. S1). By contrast, DRT2 expression resulted in a more than 100-fold reduction in burst size compared to an empty vector control, suggesting infected cells could no longer produce viable virions (fig. S1). DRT2 also allowed cell growth in liquid culture at low T5 multiplicities-of-infection (MOIs) but not high MOIs (fig. S1). This effect of DRT2 expression was not observed when the RT active site was mutated. By live-cell microscopy we observed that DRT2 expression prevents or slows phage-induced cell lysis (fig. S1). These results suggest that DRT2 expression restricts T5 propagation but does not allow individual cells to survive T5 infection, which is consistent with DRT2 functioning as an abortive infection system (14).

### DRT2 reverse transcriptase produces concatemeric cDNA upon phage infection

We hypothesized that the RT component of DRT2 would reverse transcribe the associated ncRNA upon phage infection. To test this, we infected DRT2-expressing *E. coli* with T5 phage, extracted DNA by alkaline lysis to deplete genomic DNA, and sequenced all double-stranded DNA by Tn5 tagmentation (Fig. 1A, fig. S2). We found high read coverage of a 120-bp central region (hereafter “template region”) of the 280-nt ncRNA (Fig. 1B). This peak was not observed in uninfected cells or when the RT active site was mutated (YCDD to YCAA). Notably, most of the sequencing reads mapping to the template were chimeric. Read ends that extended beyond the 3′ end of the template did not map to the downstream ncRNA, but instead mapped back to the 5′ end of the template. Similarly, read ends that extended beyond the 5′ end of the template mapped to the template 3′ end (Fig. 1B). Such read structure suggests that, upon phage infection, the DRT2 RT protein reverse transcribes the template region of the ncRNA into a cDNA comprising tandem repeats of the template region (Fig. 1C). To test this, we used a PCR assay with outward-facing primers for the template region. These primers should not amplify the template from the ncRNA-encoding plasmid, but should amplify the cDNA repeat junctions (Fig. 1D). No PCR product was visible from uninfected cells, but infection with T5 phage resulted in a PCR product of the expected size. RT activity was required, since mutating the active site of the DRT2 RT (YCDD to YCAA) led to loss of PCR product. A positive control amplicon from the DRT2 expression plasmid was readily detectable in all conditions (Fig. 1D). By quantitative PCR (qPCR), we found that uninfected cells have trace amounts of cDNA containing repeat junctions, but that T5 infection triggers a steady increase in detectible repeat junctions, plateauing after 40–60 minutes at a 1000–3000-fold increase in repeat junctions compared to an uninfected control (Fig. 1D).

We wanted to investigate the strandedness of the repetitive cDNA, since our ability to detect it by tagmentation (Fig. 1B) implied that at least some of it is double stranded (15). We performed strand-specific sequencing on DNA extracted from uninfected and T5-infected DRT2-expresing *E. coli* (Fig. 1E, fig. S2) (16). In uninfected cells we found low levels of repetitive cDNA that was mostly complementary to the ncRNA template (“minus stranded”) (Fig. 1F,G). This repetitive cDNA was not detectable after S1 nuclease treatment, indicating it is single-stranded (Fig. 1F,G). In infected cells we detected greater levels of repetitive cDNA, but the reads were also mostly minus-stranded. However, this cDNA seemed resistant to S1 nuclease: instead S1 treatment increased the number of cDNA reads (after normalization) and notably the reads were an equal mixture of plus- and minus-stranded (Fig. 1F,G). This is consistent with at least some of the repetitive cDNA in infected cells being double stranded, with S1 nuclease treatment required to make the plus-strand accessible to sequencing. Note however that this sequencing protocol is biased towards shorter sequences, which are more readily amplified and clustered on sequencing flow cells. Therefore, it may overestimate single-strand DNA abundance and underestimate double-strand DNA abundance, as these probably skew towards shorter and longer sequences respectively.

Finally, the observed chimeric sequencing reads and junction PCR products could potentially be indicative of a circular DNA species, rather than tandem repeats. To test this, we performed PCR-free long-read sequencing of total DNA from DRT2-expressing *E. coli* after T5 infection. We readily detected reads containing tandem repeats of the template region. Most commonly these reads contained 5 repeats but could contain over 100 repeats with the DNA length exceeding 12 kb (fig. S2). Together, these results suggest that phage infection triggers the DRT2 system to synthesize a repetitive double-stranded cDNA containing 120 bp tandem repeats. We name this DNA species “concatemeric cDNA”, or “ccDNA”.

### Concatemeric cDNA formation reconstitutes a promoter

**Fig. 2.**
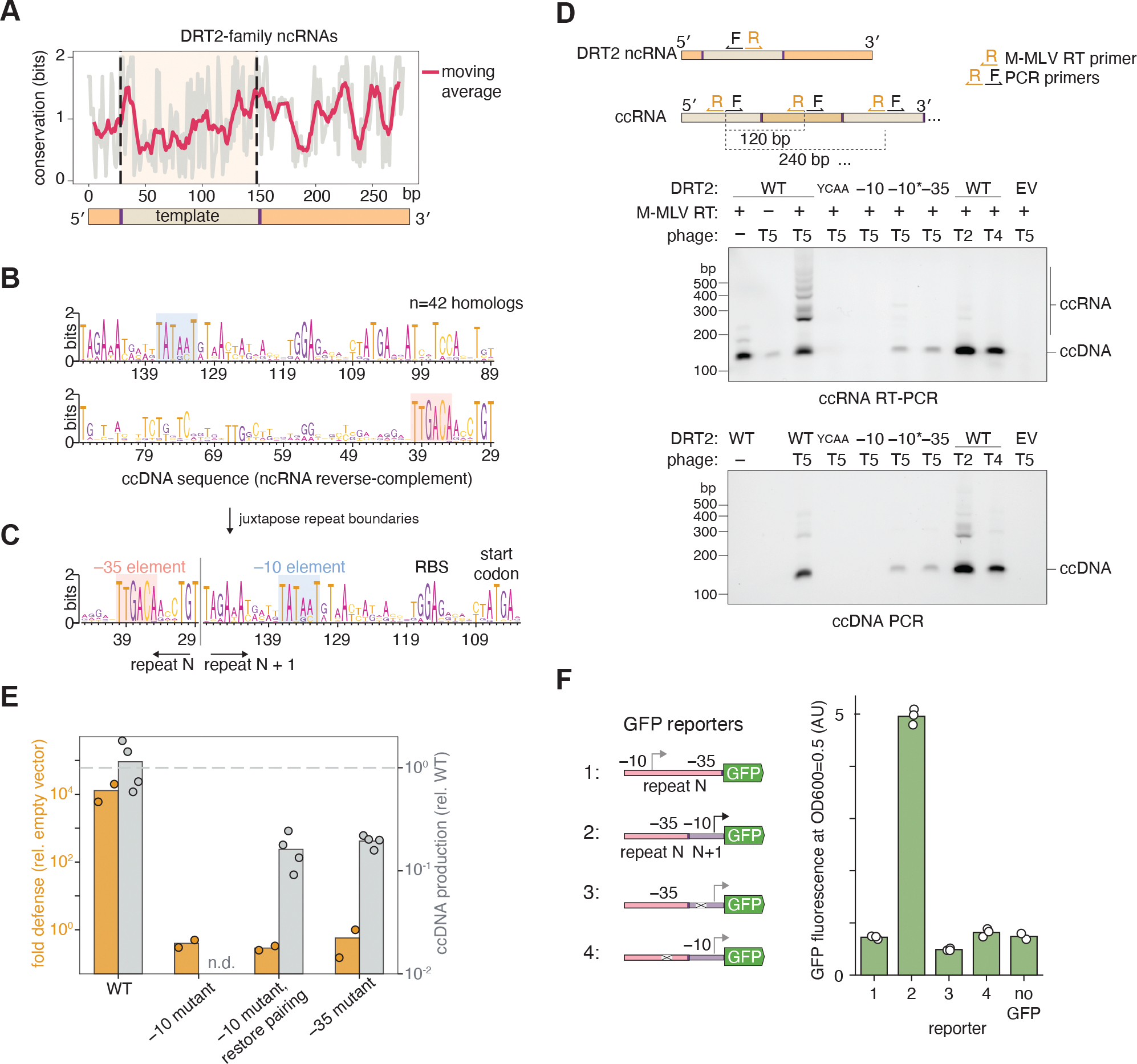
ccDNA reverse-transcribed by DRT2 can be transcribed into RNA. **(A)** Conservation at each position for 44 homologs of the *K. pneumoniae* DRT2 ncRNA. Conservation was calculated as the difference in entropy from maximum entropy at each position. The tan shaded background represents the template region. The moving average was calculated with a window size of 10 bp. (**B)** Sequence logo derived from an alignment of 42 homologs of the DRT2 ncRNA template region (shown in reverse complement to the ncRNA sequence). (**C)** The same sequence logo rearranged to correspond to a ccDNA repeat junction. Putative conserved promoter elements are shaded. (**D)** ccDNA assay by PCR, and RT-PCR assay to test for transcription of the ccDNA. WT, wild-type DRT2 system; YCAA, reverse-transcriptase active site mutant of DRT2 system; –10, mutation of the putative –10 sequence from TATAAT to CGCGGC; –10*, the same mutation combined with mutation of ncRNA bases _24_AUU_26_ to _24_GGC_26_ to restore pairing with the mutant –10 sequence; –35, mutation of the putative –35 sequence from TTGACA to AAAGGC; EV, empty vector control. (**E)** T5 defense (fold reduction in PFUs) and T5-induced ccDNA production (qPCR normalized to WT) for each of the promoter mutants. (**F)** GFP reporter assay. GFP production was assessed as the background-corrected GFP fluorescence when cells reached OD_600_=0.5. Reporter 1 contains the reverse-complement of the 120-bp ncRNA template region upstream of the GFP start codon. Reporter 2 has the same repeat permuted as found in ccDNA leading up to the start codon. Reporters 3 and 4 are identical to 2 but with the –10 or –35 mutants described above. Fluorescence was determined for three independent replicates.

Given that DRT2 functions by abortive infection, we hypothesized that the ccDNA produced in DRT2-expressing cells upon phage infection was important for preventing cell growth, and first tested if the ccDNA sequence is important for this effect. By aligning 42 closely related DRT2 ncRNA sequences, we noticed that despite high overall RNA conservation, the template region (encoding the ccDNA) tended to have the lowest nucleotide-level conservation (Fig. 2A and fig. S3). Exceptions included two conserved “ACA” trinucleotides that flanked the template, the first of which is part of the template itself (fig. S2 and S3). We made tiled transversion mutations throughout the template region and found that most mutations abolished phage defense while generally preserving ccDNA production, as determined by qPCR (fig. S3). Therefore, the ccDNA sequence itself appears to be an important determinant of phage defense.

We next inspected ccDNA sequence alignments (reverse-complement of the template region) and noticed two conserved sequences: a TATAAT motif 12 nt from the 5′ end of the template and a TTGACA motif 12 nt from the 3′ end of the template (both in the reverse-complement sense) (Fig. 2B). These motifs match the –10 and –35 elements of a σ^70^ promoter, respectively (TATAAT and TTGAAA consensus in *E. coli*) (17). The –10 and –35 elements are typically found 12-bp and 35-bp upstream of transcription start sites, with a 17-bp space between them. The potential –10 and –35 elements within the DRT2 template region, as found on the genome or within a single repeat of the ccDNA, are positioned out of order and with 91-bp spacing, so are unlikely to form a functional promoter. However, the adjacent repeats within the ccDNA would juxtapose a –10 element from one repeat with a –35 element from the next repeat with precise 17-bp spacing, reconstituting a perfect match to a strong promoter (Fig. 2C). This implies that the repetitive ccDNA produced after phage infection may template transcription of a similarly repetitive RNA, distinct from the original ncRNA species. To test this, we performed reverse transcription-PCR (RT-PCR) on RNA extracted from *E. coli* before and 30 min into T5 infection, with primers flanking the template junctions. A laddered PCR product was formed in a T5-dependent manner (Fig. 2D). To ensure the product was not residual ccDNA, we showed it only forms when M-MLV-RT was in the RT-PCR reaction. This product also depended on the integrity of the DRT2 RT active site. Mutating the –10 and –35 elements eliminated phage defense, but the –10 mutation also reduced ccDNA production (Fig. 2E). Hypothesizing that the –10 mutation disrupted base pairing of this region of the template to a region of the ncRNA upstream of the template (see below for full secondary structure), we introduced compensatory base pairs to restore this “template hairpin” and found that this partially rescued ccDNA production with the –10 mutation, but did not confer phage defense (Fig. 2D,E). Moreover the –35 mutant and rescued –10 mutant failed to produce repetitive RNA detectable by RT-PCR after T5 phage infection, suggesting these mutations disrupted the ccDNA promoter (Fig. 2D). We also placed the 120-bp sequence of a concatemerized repeat unit upstream of the start codon of a GFP reporter and monitored fluorescence over cell growth. We found constitutive expression dependent on the –10 and –35 motifs, but not when the promoter was rearranged as found in a non-concatemerized template, demonstrating that only a concatemerized repeat unit can drive gene expression (Fig. 2F).

We had noticed that the DRT2 system fails to provide defense against T2 and T4 phages, yet T2 and T4 both trigger production of ccDNA. We tested if lack of defense was due to lack of ccDNA transcription, and indeed found that T2 and T4 do not trigger repetitive RNA production detectable by RT-PCR (Fig. 2D). T4 redirects host RNA polymerase to its own middle-expressed genes by sigma appropriation, where the host σ^70^ sigma factor is altered to no longer recognize the –35 element (18). This mechanism may explain the lack of defense against T4. Together, these results show the ccDNA reconstitutes a promoter and is transcribed to generate a repetitive RNA. Promoter reconstitution across a repeat junction is reminiscent of promoter reconstitution observed in circular transposition intermediates of some DNA transposons (19, 20).

### ccDNA encodes a toxic ORF

**Fig. 3.**
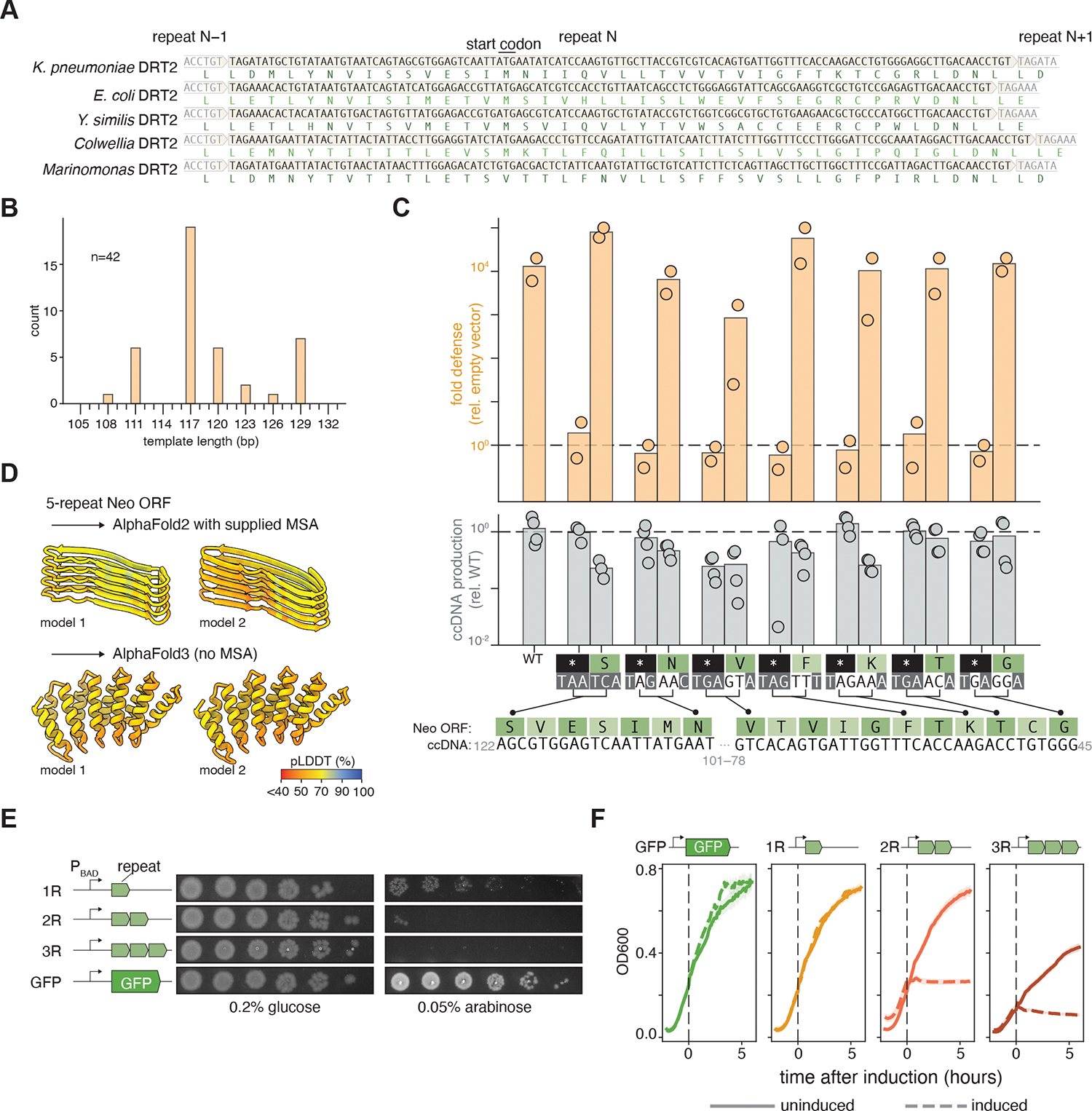
DRT2 ccDNA contains a toxic open reading frame. (**A**) Example open reading frames for five different DRT2 orthologs, all of which span theoretical repeat junctions. (**B**) Distribution of putative template lengths for 42 DRT2 ncRNA orthologs. Major x-axis ticks are for multiples of three. **(C)** The indicated ccDNA codons were either mutated to stop codons (asterisks) or synonymous codons as indicated. The graphs show T5 defense (fold reduction in PFUs) and T5-induced ccDNA production (qPCR) for each of the codon mutants, compared to a wild-type control. (**D**) Protein structure prediction for 5 repeats of the *K. pneumoniae* DRT2 Neo ORF. For AlphaFold2 a multiple-sequence alignment (MSA) of 42 Neo orthologs was supplied. No MSA was used for AlphaFold3. Two representative models are shown for each prediction. (**E)** Inducible expression of 1–3 repeat units of the Neo protein leads to toxicity. 10-fold dilutions of exponentially growing *E. coli* were plated on repressive media (0.2% glucose) or inducing media (0.05% arabinose). P_BAD_, arabinose-inducible promoter. (**F)** The same constructs as in **E**, grown in LB for two hours before induction with 0.05% arabinose. Solid lines represent uninduced controls. Shading represents the standard error of three replicates.

We next tested if the transcribed ccDNA could encode a protein. From 42 close homologs of the *K. pneumoniae* DRT2 system we inferred theoretical ccDNA sequences by extracting, reverse-complementing, and concatemerizing the sequences between the two conserved “ACA” motifs, including the first of these motifs. All theoretical ccDNA sequences contained a single reading frame that spanned repeat junctions and lacked any stop codons (Fig. 3A, fig. S4). Furthermore, all repeat units were of nucleotide lengths divisible by three (e.g., the *K. pneumoniae* repeat is 120 nt, most repeats were 117 nt, several were 111 nt or 129 nt) (Fig. 3B). These observations suggest that it is important that the ccDNA contains a reading frame that spans multiple repeats, as a repeat unit not divisible by three would result in a frameshift in the next repeat unit and therefore include a stop codon. Supporting this idea, a conserved potential ribosome-binding site and start codon can be found 13-nt downstream of the –10 promoter motif (Fig. 2C). We predicted that, if the coding capacity of the ccDNA is important, introducing a stop codon into the ncRNA template would abolish phage defense, whereas synonymous mutations (nucleotide substitutions that preserved the coded amino acid) would be neutral. We tested this at seven codons throughout the ncRNA template that displayed low nucleotide-level conservation (to prevent disrupting ccDNA production). At all seven positions, we found that synonymous codons allowed phage defense at levels identical to wild type, whereas stop codons entirely abolished defense (Fig. 3C). All mutations preserved ccDNA production. Therefore, encoding an open reading frame in the ccDNA is important for phage defense. Because this gene is only synthesized in phage-infected cells and could potentially be very long (depending on the ccDNA repeat count), we refer to it as *neo* (nearly-endless ORF).

The Neo protein sequence does not match any annotated protein domain. Most conserved amino acids in the Neo repeat unit can be attributed to nucleotide-level conservation of the promoter and ACA elements of the ccDNA. However, the repeat unit has a strong bias towards hydrophobic codons (fig. S4). Protein structure prediction of a five-repeat Neo protein using AlphaFold2 (21) or AlphaFold3 (22) yields low-confidence repetitive arrays of beta strands or alpha helices (Fig. 3D), which do not imply any clear enzymatic activity. Nevertheless, given that DRT2 functions by abortive infection, we hypothesized that *neo* expression could result in cellular dormancy. We cloned one to three 120-nt (or 40 amino acid) *neo* repeat units downstream of an arabinose inducible promoter. In this experiment, the repeats were synonymously recoded from the natural ccDNA sequence to remove the internal –10 and –35 promoter elements, and the final repeat was terminated with a stop codon. We transformed these *neo* constructs into *E. coli* and assayed for cell growth on media containing arabinose (inducing), compared with growth on glucose, which represses the promoter. Relative to a GFP control, expression of a single repeat reduced colony counts and reduced colony size. This effect was exacerbated with two repeats, while with three repeats no colonies grew on inducing media (Fig. 3E). We were unable to clone four or more repeats due to toxicity; all sequenced colonies contained fewer repeats or premature stop codons. To test if *neo* expression stops cell growth, we induced one- to three-repeat Neo constructs in liquid cultures. Induction did not affect cell growth of one-repeat constructs compared to a GFP control, but the optical density plateaued immediately after induction of two-repeat *neo*, and it declined after three-repeat *neo* induction (Fig. 3F). This suggests that *neo* expression arrests cell growth. Overall, these results demonstrate that the coding capacity of the ccDNA is important for phage defense, and that the encoded Neo can restrict cell growth when expressed.

### Molecular basis for ccDNA synthesis

Our results demonstrate that upon phage infection, the DRT2 RT reverse transcribes the template region of the DRT2 ncRNA into a concatemeric repetitive ccDNA that then templates transcription (“forward transcription”) of the ccDNA into a repetitive *neo* mRNA that is then translated into a toxic Neo protein. How does the DRT2 RT recognize the DRT2 ncRNA and select the template region? What is the primer for DNA synthesis? Furthermore, our model requires transcription of the ccDNA, which is only possible for double-stranded DNA as the *neo* gene is on the “antisense strand” relative to the sense of the original ncRNA. How does the DRT2 RT produce double-stranded DNA?

**Fig. 4.**
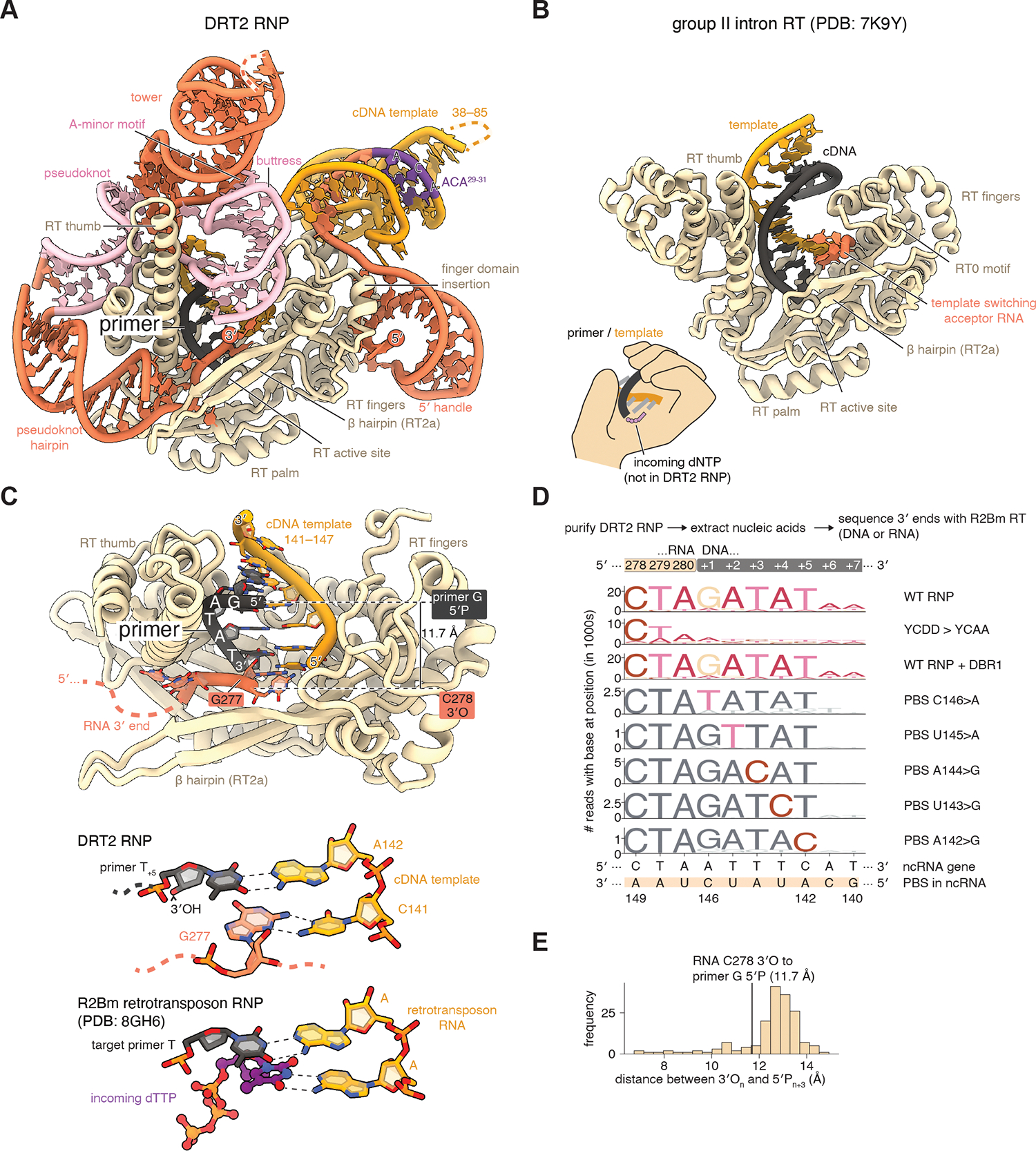
Structure of the DRT2 ribonucleoprotein complex. **(A)** Cryo-EM structure of the DRT2 reverse transcriptase bound to the DRT2 ncRNA. Dashed lines represent nucleotides not resolvable in the cryo-EM density. (**B**) Structure of a group II intron-encoded RT in the same orientation as **A** (23). (**C**) The DRT2 RNP contains a 5 nt DNA primer in the active site. The inset shows how DRT2 G277 occupies the same position as the incoming thymidine triphosphate (dTTP) in a stalled elongating RT enzyme (57). (**D**) Nucleic acids extracted from DRT2 RNPs were sequenced using Ordered Two-Template Relay (24). Reads were aligned to the 3′ end of the DRT2 RNP and sequence logos calculated. PBS mutants derive from a library of PBS mutant sequences, see fig. S6 for all PBS mutations. YCDD>YCAA, RT active site mutation; DBR1, yeast debranching enzyme; PBS, primer-binding site (**E**) Histogram of distances between 3′ oxygens and 5′ phosphates for RNA bases separated by two intervening nucleotides, calculated from the DRT2 ncRNA structure itself. Dotted line indicates the distance between the last observed base of the DRT2 ncRNA and the first observed base of the primer (also shown in **C**).

To address these questions, we overexpressed the DRT2 operon from a T7 promoter in non-phage-infected cells and purified the DRT2 RT using a Strep-II tag on the protein C-terminus. The protein co-purified with stoichiometric quantities of an RNA of size consistent with the 280-nt DRT2 ncRNA. We imaged this ribonucleoprotein (RNP) complex by cryogenic electron microscopy (cryo-EM) and solved its structure at 2.9 Å resolution (Fig. 4A, fig. S5). This resolution allowed unambiguous assignment of the RNA density as the DRT2 ncRNA, indicating that in non-infected cells, the DRT2 RT and ncRNA form a complex. The DRT2 RT protein adopts the conventional ‘right-hand’ fold of a group II intron family RT, with two unique elements being a particularly long alpha-helical thumb domain, and a unique insertion in the fingers domain above the loops important for template capture during template jumping of the group II intron RT (RT0 motif) (Fig. 4B) (23). These specializations allow specific binding to the DRT2 ncRNA. The region of the ncRNA 5′ to the template (“5′ handle”) forms a hairpin that docks into the finger-insertion domain. The 3′ region of the ncRNA downstream of the template is intricately folded and intimately associated with the elongated thumb domain of the RT. One long 34-nt hairpin (the “tower”) and a shorter 18-nt hairpin (the “buttress”) form A-minor motif interactions and rise against one side of the thumb, an interaction which is secured by a potassium ion-stabilized pseudoknot that wraps around the other side of the thumb. Disruption of any of these features of the ncRNA, with the exception of shortening the tower hairpin, led to loss of ccDNA production upon phage infection and loss of phage defense activity (fig. S3). The rest of the template region is loosely bound to the RT, as evidenced by weaker cryo-EM density, but template bases 29–37 and 86–135 are visible as three helices forming a three-way junction. The looser binding of the template compared to its bracketing sequences may facilitate its repeated cycling through the RT active site.

The final eight nucleotides of the template (bases 141–148) are visible within the RT active site. Strikingly, template bases 142–146 are already annealed to a 5-nt DNA primer “GATAT,” we therefore refer to these template bases as the primer-binding site (PBS) (Fig. 4C). To test if the primer was part of a longer DNA molecule not visible in the cryo-EM density, we made use of the template jumping and DNA- and RNA-dependent DNA polymerase activities of the *Bombyx mori* non-LTR retrotransposon RT to sequence all nucleic acids associated with our purified DRT2 RNPs (24) (Fig. 4D, fig. S6). Most of the reads mapped to the ncRNA, but surprisingly, the majority showed a “GATAT” sequence directly after A280 at the 3′ end of the ncRNA, a mismatch to the ncRNA gene but an exact match to the primer sequence (Fig. 4D). The presence of this extension is dependent on the DRT2 RT active site, and its sequence is templated by the PBS since mutations in the PBS resulted in complementary changes to the extension (Fig. 4D, fig. S6). We conclude that the “GATAT” DNA primer is covalently linked to the 3′ end of the ncRNA and is synthesized by the DRT2 RT. Further PBS mutations and alkaline hydrolysis support our assignment of the “GATAT” bases as DNA and the preceding bases as RNA (fig. S6). The primer was resistant to yeast debranching enzyme DBR1 (Fig. 4D), suggesting it is connected by a 3′-5′ phosphodiester bond to the ncRNA A280. The 5′ phosphate of the primer G and the 3′ oxygen of the ncRNA C278 (the last RNA base clearly resolved in the cryo-EM density) are separated by 11.7 Å, which would readily be bridged by two additional RNA bases (Fig. 4E).

**Fig. 5.**
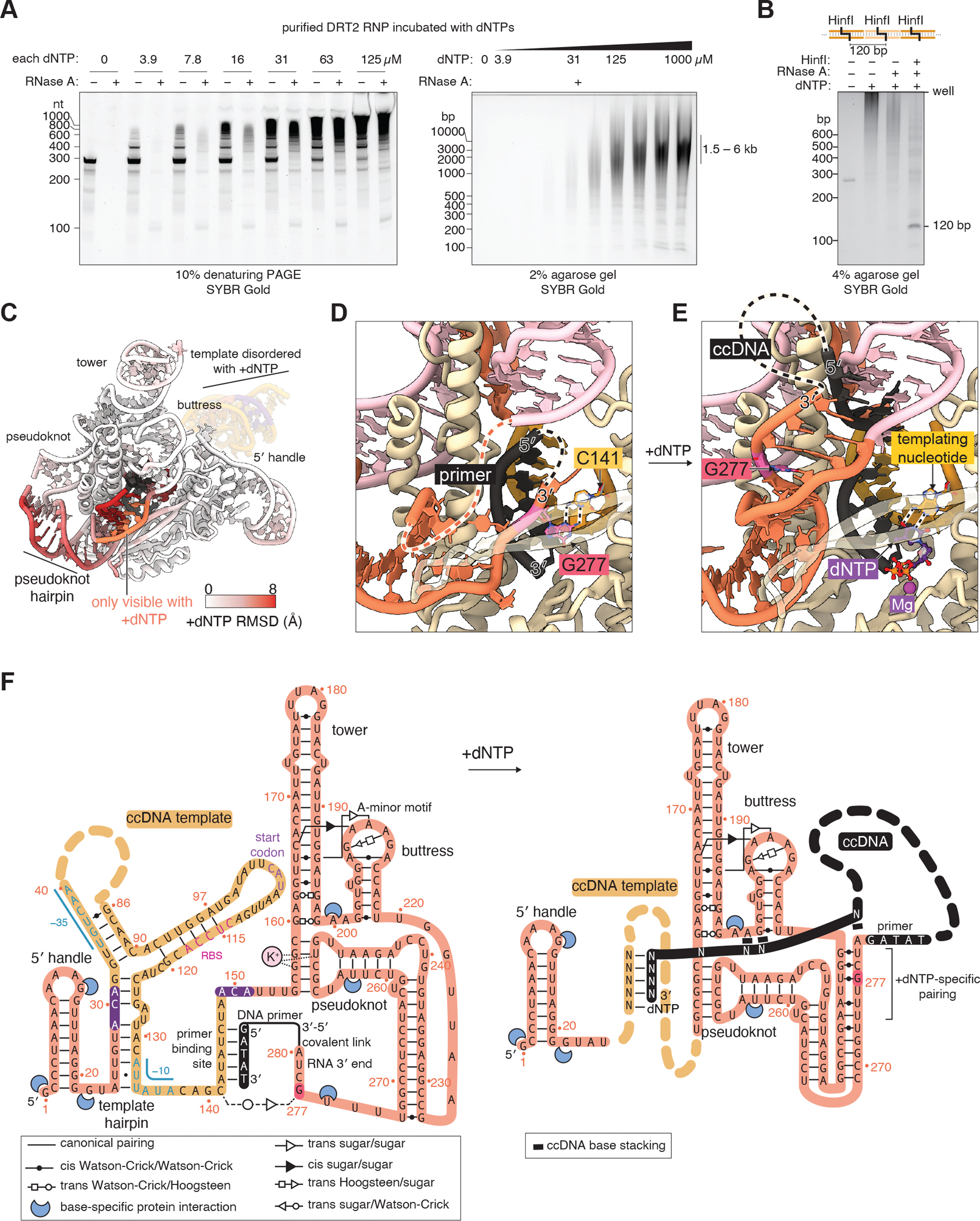
Structure of DRT2 in the elongating state. **(A)** The purified DRT2 RNP was incubated with deoxynucleotide triphosphates (dNTPs) at the indicated concentrations, and products were visualized by denaturing gel electrophoresis (left) or agarose gel electrophoresis (right). In the agarose gel each lane has 2-fold more dilute dNTPs compared to the neighbor on the right, except lane 1 has no dNTPs. The indicated concentrations are of each dNTP. (**B)** Each ccDNA repeat contains a natural HinfI restriction endonuclease site (staggered lines). Purified in vitro ccDNA products were incubated with HinfI and or RNase A. (**C**) Structure of the DRT2 RNP in the elongating state. Residues are colored by the root-mean-square deviation (RMSD) of their positions compared to the resting state. The template region is not well-resolved in the elongating state and is shown for reference as a transparent background. **(D)** Details of the DRT2 reverse transcriptase active site in the resting state or (**E**) in the elongating state (with dNTPs). (**F)** Secondary structure diagram of the DRT2 ncRNA in the resting and elongating states. Non-canonical base pairs are notated using the nomenclature of Leontis and Westhof (64).

RT enzymes elongate primers by transesterification of a deoxynucleotide triphosphate (dNTP) complementary to the template RNA base next to the PBS, which for the DRT2 RNP is base C141. Instead, we saw that the incoming dNTP binding site is blocked by a guanosine nucleotide from the 3′ end of the ncRNA (G277) which forms a non-Watson-Crick pair with template base C141 (Fig. 4C). We thought that G277 might inhibit the RT activity of the DRT2 RNP, so incubated the purified DRT2 RNP with varying concentrations of dNTPs and assayed for cDNA production on denaturing and agarose gels. Instead, we observed formation of a laddered DNA species, the average size of which increased with higher dNTP concentrations, with 1 mM dNTPs stimulating formation of a 1.5–6 kb DNA product (Fig. 5A). At 4–16 *μ*M dNTPs the smaller products were more readily observed, with their mobility on a denaturing gel being perfectly consistent with the 120 nt increments observed in ccDNA produced in vivo, and moreover these shifted in size after RNase A treatment in a manner consistent with a covalent connection between the primer and ncRNA (Fig. 5A). The DRT2 template region naturally contains a GANTC HinfI restriction endonuclease recognition site, and when we treated the cDNA products with both RNase A and HinfI, a 120-bp product was formed, identical in size to the repeat unit of the ccDNA produced in vivo (Fig. 5B). Because HinfI is specific to dsDNA, this observation also suggests that the cDNA produced in vitro is mostly double-stranded. Therefore, purified DRT2 RNPs are active in producing ccDNA in the absence of phage-specific cues.

We next made cryo-EM grids of a DRT2 RNP that was frozen 2.5 min after addition of 1 mM dNTPs (fig. S7). The imaged particles were in a mixture of states that mostly resembled the “resting” state in the absence of dNTPs, but in 11% of high-quality particles we observed a distinct conformation that we term the “elongating state” (Fig. 5C, fig. S7). We solved the structure of this state at 3.2 Å resolution and observed an incoming dNTP molecule in the RT active site (Fig. 5D,E), displacing G277. G277 and five neighboring bases from the ncRNA 3′ end instead form six base pairs with the linker between the pseudoknot and buttress (Fig. 5E,F). This linker was disordered in the resting state of the DRT2 RNP (Fig. 5C). Consistent with the elongating state containing ccDNA, we observed that the active site cDNA was extended compared to the resting state primer, with the nascent ccDNA 5′ end exiting the RNP through an “exit channel” formed by the G277-linker duplex and maintaining proximity to the ncRNA 3′ end, further consistent with their covalent connection. The ncRNA secondary and tertiary interactions in the resting and elongating states are summarized in Fig. 5F. We suggest that the exit channel promotes melting of the nascent ccDNA from the RNA template, preventing RNase-H-mediated cleavage of the template and thereby facilitating template recycling.

**Fig. 6.**
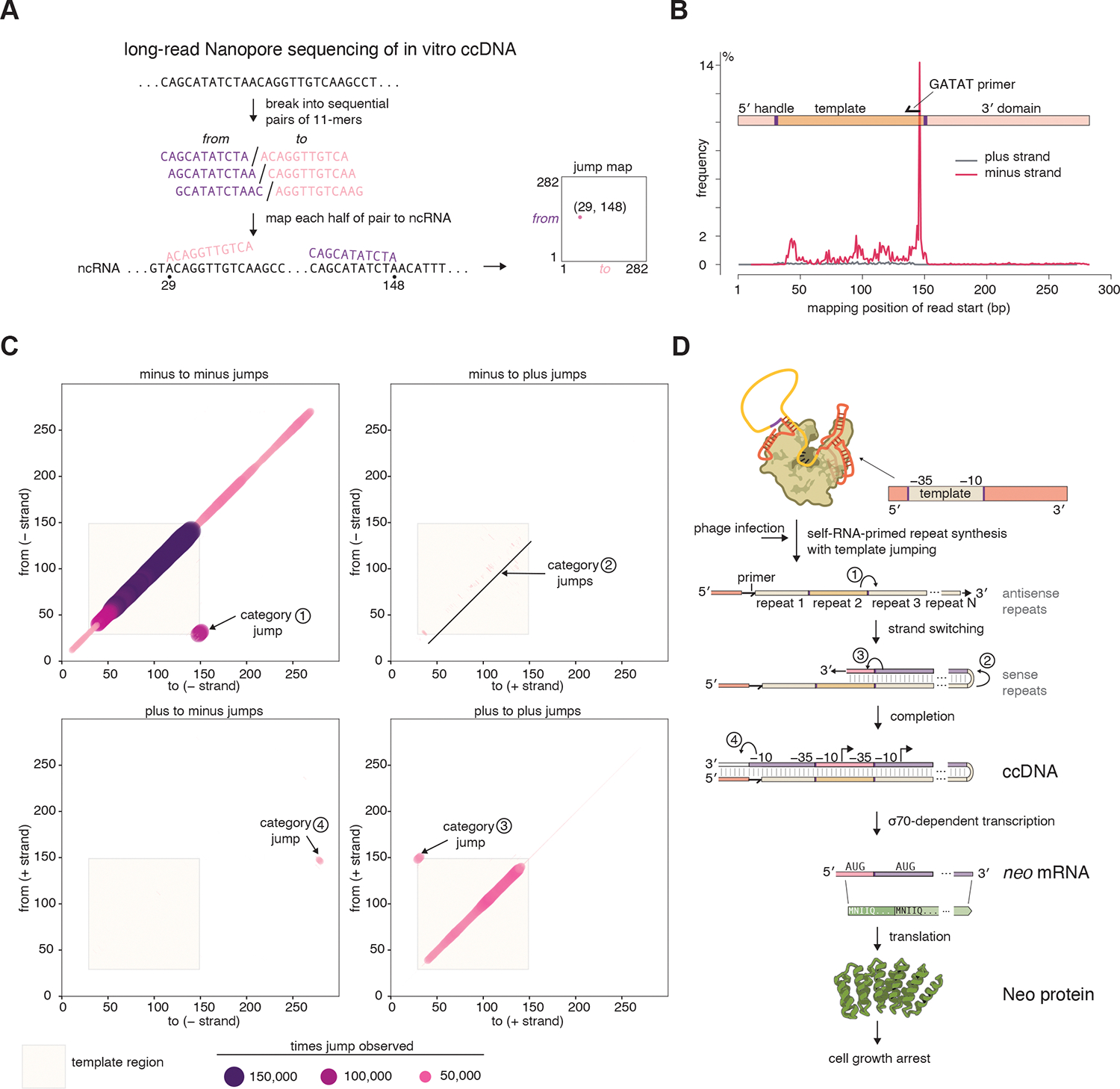
Molecular mechanism of ccDNA synthesis. (**A**) Purified in vitro ccDNA products were sequenced by Nanopore long-read sequencing, and reads were analyzed as sequential 11-mers (the minimum k-mer length for unique mapping to the DRT2 ncRNA). Jump maps were generated by plotting pairs of neighboring mapped 11-mers. The position on the y-axis corresponds to the mapped position of the 3′ base of the 5′ 11-mer, the position on the x-axis corresponds to the mapped position of the 5′ base of the 3′ 11-mer. Off-diagonal points represent jumps, or chimeric reads. (**B**) Distribution of mapping positions of the first 11-mer on the Nanopore read after the sequencing adapter. The most frequent mapping position corresponds to the “GATAT” primer. (**C**) Jump maps for in vitro ccDNA. Each point is sized and colored according to the observed frequency of that jump in all reads. The shaded tan background corresponds to the template region of the ncRNA. Windows mapping to the same strand as the ncRNA are called plus-strand windows; windows mapping to the antisense of the ncRNA (as expected from reverse transcription) are called minus-strand windows. Four categories of jump are labelled. “Category 1” jumps are the minus-to-minus strand jumps between the start and end of the template region, as observed in our other sequencing data, and account for 65% of all off-diagonal jumps. “Category 2” jumps are minus-to-plus strand reversals and likely represent the DRT2 RT switching direction. These collectively account for 11% of off-diagonal jumps. “Category 3” jumps are plus-to-plus across the repeat junctions, likely representing “second-strand synthesis” and account for 17% of off-diagonal jumps. “Category 4” jumps are plus-to-minus and likely represent read-through from the primer back into the ncRNA. These account for 7% of off-diagonal jumps. (**D**) Model for the mechanism of the DRT2 defense system. The jump categories from **C** are labelled.

We wanted to more deeply understand the nature of the cDNA synthesized by the DRT2 RNP in vitro. We directly sequenced the in vitro-produced, RNase-treated ccDNA by Nanopore sequencing (Fig. 6A, fig. S8). From 119,787 reads which had the expected positions of the adapter sequences, we first observed that most reads contained the expected pattern of consecutive repeats antisense to the ncRNA template region, with the majority of reads starting at the PBS with the “GATAT” sequence corresponding to the DNA primer observed in the cryo-EM density (Fig. 6B, fig. S8).

To quantify all template jumps captured by the reads, we split each Nanopore read into sequential pairs of 11-mers (the minimum length unique k-mer) and mapped each half of each pair to the ncRNA sequence (both sense and reverse-complemented) (Fig. 6A). The resulting “jump map” confirms that the most common jump (“Category 1”) is from minus-strand base 29 to minus-strand base 148, similar to the in vivo sequencing data (Fig. 6C). The equivalent jump was also frequently seen for the plus strand (“Category 3”), showing that both plus- and minus-strand tandem repeats are found. There were two other prominent jump categories. One jump is from base 31 of the minus strand, to base 34 of the plus strand. Similar minus-to-plus transitions can also be seen throughout the template region but are more common at its 5′ end (Fig. 6C). These jumps – “Category 2” jumps – imply the DRT2 RT can change direction during reverse transcription and switch from using the DRT2 ncRNA as a template to using the previously synthesized cDNA, becoming a DNA-primed DNA polymerase (Fig. 6C). Reads having such (minus-strand repeat)_x_ (plus-strand repeat)_y_ structure almost never have more plus-strand repeats than minus-strand repeats (y <= x), consistent with the minus-strand repeats templating the plus-strand repeats (fig. S8). Such reads containing tandem minus-strand repeats followed by tandem plus-strand repeats likely correspond to an elongated DNA hairpin that behaves as dsDNA, accounting for the sensitivity of the cDNA to HinfI (Fig. 5B) and likely explaining why S1 nuclease was required to sequence plus-strand repeats in DNA extracted from cells (Fig. 1F). The final jump category (“Category 4”) is from the primer-binding site part of the template plus strand to the 3′ of the ncRNA minus strand (Fig. 6C). This is further evidence for the covalent primer-ncRNA connection and likely corresponds to read through of the DRT2 RT back into the primer itself and into the 3′ domain of the ncRNA (Fig. 6D). Given the importance of the 3′ domain of the ncRNA for ccDNA synthesis (fig. S3), this may lead to RNP disassembly.

Taken together, these structural, biochemical, and sequencing-based observations suggest that the DRT2 RNP performs RNA-primed reverse transcription of the template region, which is loosely held between a rigid 5′ and 3′ scaffold. Stochastically during minus-strand repeat synthesis, the DRT2 RT switches from RNA-templated to cDNA-templated DNA polymerase activity to perform second-strand synthesis. The resulting ccDNA is an elongated hairpin that functionally acts as dsDNA (Fig. 6D).

## Discussion

We have discovered a mechanism by which DRT2 RTs provide phage defense (Fig. 6D). The DRT2 RT constitutively binds its associated ncRNA and synthesizes a short covalently-attached DNA primer. Upon phage infection, the RT initiates reverse transcription of the central template region of the ncRNA and continually jumps to the start of the template after reaching the end, producing a long repetitive ccDNA that then becomes double-stranded. The repeat junctions in the ccDNA reconstitute a promoter and long repetitive ORF (*neo*), resulting in transcription of a repetitive mRNA and translation of the repetitive Neo protein, the toxicity of which increases with the number of repeats it contains. This likely results in cell growth arrest and inhibition of phage propagation. This mechanism of amplification of a single template into a concatemeric DNA is conceptually similar to rolling circle amplification (RCA) from DNA polymerase activity on circular DNA templates (25), but using a linear RNA template instead.

RTs are well known as exceptions to the unidirectional flow of genetic information from DNA to RNA to protein, most commonly to allow RNA-based mobile genetic elements to be stored or propagated as DNA (1). Reconstitution of a gene by DRT2 is a startling extension of this concept: DNA lacking direct coding information is transcribed into a “non-coding” RNA, which is reverse transcribed back to a new DNA that gains coding capacity, which is then transcribed to a mRNA and then translated. Production of an ORF not directly present in DNA is reminiscent of RNA splicing, where non-coding introns must be removed from a pre-mRNA for the RNA to have a coherent reading frame for translation (26, 27). Incidentally, the most conserved protein in the eukaryotic spliceosome is the reverse transcriptase domain protein Prp8, although catalysis is carried out by RNA (3, 28).

The activity of the DRT2 RT is also reminiscent of another domesticated eukaryotic reverse transcriptase: telomerase. Telomerase extends telomeres by adding 4–8 nt ssDNA tandem repeats to the 3′ end of chromosomal DNA (29). The template for these tandem repeats is a large ncRNA that contains the template 3′ to a stem and 5′ to a conserved pseudoknot, identical in concept to the DRT2 ncRNA except that the DRT2 template is the much larger 120 nt (fig. S9). Telomerase has repeat addition processivity (RAP) where arrays of repeats are synthesized without substrate dissociation. For human telomerase, the telomerase RNA template region has a full repeat sequence followed by a partial repeat termed the alignment region: 5′-CUAACCCUAAC-3′ (full repeat underlined). The alignment region anneals to the telomeric DNA 5′-GTTAG-3′, and the repeat templates extension with 5′-GGTTAG-3′, which then melts and reanneals with the alignment region to repeat the cycle (30). We speculate that repeat synthesis by DRT2 may also involve RAP activity, and that an analogous role to the alignment region may be provided by the conserved “ACA” sequences that flank the template. In this model, the DRT2 template 5′-ACA…CA-3′ anneals to the ccDNA 5′-...TGT-3′ and is extended in a similar fashion. How intervening “ACA” sequences present within the template would not interfere with repeat synthesis remains to be determined, but may involve the close proximity of the template-flanking “ACA”s to tightly bound RNA elements (the 5′ handle or pseudoknot) stalling the reverse transcriptase. We suggest that the conceptual similarities between DRT2 and telomerase are due to convergent evolution: the pseudoknots have different 3D structures and engage the RT protein in different ways (31) (fig. S9), and phylogenetically telomerase is most closely linked to non-LTR retrotransposon RTs in eukaryotes (32). Instead, we think that DRT2 represents a second instance of RT activity being repurposed for repetitive DNA synthesis, with quite different biological consequences.

Why does the DRT2 system employ such an unconventional mechanism to produce a coding RNA? One reason could be the extreme toxicity of the Neo protein. Our data suggest that the lengths of Neo proteins produced in vivo would commonly be five repeats and in some cases over 100 repeats (fig. S2), whereas we could only successfully clone up to three Neo repeats under the control of the tightly repressible araBAD promoter. Hiding the *neo* gene as a non-transcribable permuted template within a ncRNA may provide a more stringent gate on *neo* expression, and could mitigate genomic instability from directly encoding such a repetitive gene (33). Some phages, including T5, degrade the host genome (but not RNA) before injecting their own DNA (34). In these cases, transcriptional regulation of a toxic gene might not be possible, whereas hiding the toxic gene within RNA to be converted back to DNA once host DNA degradation ceases could circumvent this problem. On the other hand, the DRT2 mechanism we characterized is reliant on host RNA polymerase and translational machinery, which are often hijacked by phage, and indeed we observed that T4 phage is able to circumvent defense by DRT2 at least partly by repressing transcription of the ccDNA (Fig. 2D). We speculate that diverse DRT2 systems might produce quite different ccDNA sequences to those characterized in this study and predict that some of these may reconstitute phage-specific promoters instead.

The arsenal of defense systems employed by bacteria to defend against phage infection is vast, with many mechanisms presently emerging (35). The majority provide a “front-line” defense by degrading phage nucleic acids, for example restriction-modification systems and CRISPR-Cas. But many recently characterized systems are instead abortive infection systems that use different mechanisms to produce cell dormancy in response to phage infection, often by phage triggering a signal that activates a toxic protein. Such signals include synthesis of chemical messengers or allosteric communication through protein domains to activate effectors (35–37). The DRT2 defense system fits into this paradigm, where the signal is gene creation and the effector is the gene product. Some abortive infection systems can directly sense phage proteins to trigger toxicity (38–40), but for many systems the triggers are unknown. We don’t yet know how phage infection triggers the RT activity of DRT2 and note that this is quite distinct from retron RTs which constitutively synthesize cDNA (41, 42). Because different phages could all trigger ccDNA production (Fig. 2D), and because the nucleotide concentration required for in vitro ccDNA synthesis by the DRT2 RT is rather high (Fig 5A), we speculate that changes in nucleotide metabolism or abundance after phage infection could be a potential trigger, but this remains to be experimentally determined.

The Neo protein produced by the DRT2 system is unlike toxins usually associated with phage defense abortive infection effectors, which frequently contain domains like nucleases or nucleoside-degrading enzymes (4). In contrast, Neo does not match any annotated protein domain, and protein structure prediction only produced low-confidence models that did not imply obvious enzymatic activity. As a gene, the ccDNA has many unusual features: inherently, every repeat unit will contain a promoter, and inherently every *neo* mRNA repeat unit will contain a ribosome binding site and a start codon. These structures could act as sponges for RNA polymerase or ribosomes, or promote ribosome or RNA polymerase collisions, on top of the inherent toxicity of Neo translation. Further, the *neo* mRNA may lack stop codons and therefore engage the tmRNA or ArfA translational rescue machinery (43). However, given our proposed extended hairpin structure of the ccDNA (Fig. 6D), it is possible that once RNA polymerase finishes transcribing the final plus-strand repeat it could read through the hairpin loop and continue transcribing the minus-strand repeats from ssDNA, which would produce stop codons in the *neo* mRNA. Further work is needed to delineate the precise mechanisms of toxicity by the Neo protein.

In conclusion, our study has revealed that prokaryotic reverse transcriptases can reconstitute repetitive genes by reverse transcription to provide phage defense. The mechanism of DRT2 introduces a new form of genomic “dark matter”: genes that only become visible after reverse transcription, and a new mode of gene regulation in prokaryotes: regulation by gene synthesis.

## Materials and Methods

### Plasmids and cloning

The full sequences of plasmids and oligonucleotides used in this study are found in Table S1. The DRT2 system is expressed by plasmid pLG010 (Addgene Plasmid #157888) (4). Mutants of pLG010 were generated by HiFi assembly (NEB) or KLD cloning (NEB) and transformation into NEB 5-alpha competent *E. coli* (NEB). For overexpression and purification, the DRT2 operon was subcloned into a pET bacterial expression vector by Gibson Assembly, placing the first nucleotide (“G1”) of the DRT2 ncRNA directly after a T7 promoter and the last codon of the DRT2 RT directly before a GS linker and Twin-StrepII tag. The Neo ORF was cloned by Golden Gate assembly of annealed oligos with unique linkers as described (44). Plasmid sequences were verified by T5 tagmentation and high-throughput sequencing, as described previously (45). Repeat-containing plasmids were further verified by long read sequencing (Primordium Labs). Antibiotics were used at the following concentrations: ampicillin 100 μg/mL and chloramphenicol (Cm) 25 μg/mL.

### Phage propagation

All phage were propagated in liquid culture. 30 mL cultures of *E. coli* BL21-AI were grown at 37°C in LB containing 1 mM MgCl_2_ + 1 mM CaCl_2_ and infected with phage at an MOI of 0.1. After lysis overnight, 500 μL of chloroform was added and debris was pelleted by centrifugation. Supernatants were stored at 4°C with another 200 μL of chloroform.

### Phage plaque assays

*E. coli* BL21-AI carrying pLG010, a pACYC empty vector control, or pLG010 variants, was grown to saturation at 37°C in LB containing Cm. 200 μL of each culture was placed on the surface of a LB + 1.5% agar plate containing Cm before pouring over 4 mL of molten (50°C) top agar (LB + 0.3% agar + Cm + 1 mM MgCl_2_ + 1 mM CaCl_2_) and swirling to mix. Phage were serially ten-fold diluted in LB and 3 μL drops were spotted onto the dried top agar. Plates were inverted, incubated overnight at 37°C, and imaged with a white backlight. The most diluted spot with countable plaques was used to quantify phage plaque forming units (PFUs); assays where the most diluted spot with plaques had too many plaques to count but the next dilution had no plaques were counted as 0.5 plaques at the next dilution. Defense activity was quantified as the number of PFUs in the empty vector control divided by the PFUs in the condition tested. Biological replicates used independently prepared T5 phage stocks on the same bacteria lawn.

### Phage adsorption assay

Phage adsorption was measured as described (46). *E. coli* BL21-AI carrying pLG010 or a pACYC empty vector control was grown at 37°C in LB containing Cm to OD_600_ = 0.6. T5 phage was added to a MOI of 0.0002. After 0 min or 30 min at 37°C, 0.1 mL aliquots were taken, diluted with 0.9 mL of 20 mM Tris pH 7.4, 100 mM NaCl, 20 mM MgCl_2_, and spun for 5 min at 20,000*g*. Ten-fold dilutions of the supernatant had phage titer determined using the soft-agar overlay method.

### Phage burst size assay

Phage burst size was determined as described (47). *E. coli* BL21-AI carrying pLG010 or a pACYC empty vector control was grown at 37°C in LB containing Cm to OD_600_ = 0.6. T5 phage was added to a MOI of 0.0002. After 15 min at 37°C, free phage were removed by centrifugation and suspending the pellet in fresh media, twice. The suspension was then diluted 10,000-fold and at the indicated time points the phage titer in the supernatant was determined.

### Live cell imaging

*E. coli* BL21-AI carrying pLG010 or pMW323 (constitutive GFP) was grown at 37°C in LB containing Cm or Ap to OD_600_ = 0.8. Cells were pelleted at 7000*g* at room temperature and washed with LB four times to remove antibiotic, before suspension in LB + 1 mM MgCl_2_ + 1 mM CaCl_2_ to OD_600_ = 8. 0.5 mL of each suspension was mixed and vortexed briefly, before addition of T5 phage to 5×10^10^ PFU/mL (estimated MOI=8) and incubation with shaking for 15 min at 37°C. 100 μL of suspension was then incubated with 0.1 mg/mL DAPI at room temperature for 10 min, before pelleting at 20000*g* for 1 min and suspension in 10 μL PBS + 0.2 μg/mL propidium iodide. 1 μL was spotted on an agarose pad (1.5% agarose dissolved in LB + 1 mM MgCl_2_ + 1 mM CaCl_2_) and covered with a glass coverslip. The sample was imaged on a Stellaris 5 confocal microscope (Leica) using a 63× objective and 2.5 AU pinhole diameter at room temperature. The same field of view was imaged every 6 – 40 min as an 8 μm z-stack (one image every 0.5 μm). For each timepoint, a maximum-Z projection was calculated using Fiji (48). For the brightfield channel a standard deviation Z projection was also calculated. The standard deviation projection was manually masked and used to train an Omnipose (49) model to segment cells from all time points. The segmentation masks were then registered over timepoints in Fiji using TrackMate (50), with feature penalties for circularity, area, and GFP intensity. Cells were then divided according to the bimodal GFP intensity distribution, and survival was calculated as the percentage of GFP+ or GFP- cells remaining relative to the first imaged timepoint.

### ccDNA sequencing by tagmentation

*E. coli* BL21-AI containing pLG010 or pLG253 (pLG010 with YCDD>YCAA mutation) was grown at 37°C in LB + Cm + 1 mM MgCl_2_ + 1 mM CaCl_2_ to OD_600_ = 0.8. Cultures were split in half: one half had DNA extracted immediately, while the other half was infected with T5 phage at 5×10^10^ PFU/mL (estimated MOI = 50) at 37°C with shaking for 30 min before DNA extraction. DNA was extracted by pelleting cultures followed by alkaline lysis and column purification (Qiagen Spin Miniprep Kit). DNA (100 ng for each sample) was mixed with 10 ng of spike-in DNA (pMW342, Table S1) and tagmented with Tn5 as previously described (45), amplified with barcoded primers, and sequenced using a MiSeq in single-end mode (250 bp read 1). Reads were trimmed and mapped to the expression plasmid, host genome, and T5 phage using BWA-MEM (51). Reads mapping to the ncRNA were broken into consecutive 11-mers (the minimum length for unique hashing) and each 11-mer was assigned to its corresponding position within the ncRNA. Mapping along each read was visualized with a custom Python script: each read is represented as a series of line segments, one segment per 11-mer, where the x coordinate is determined by the mapping positions of the longest consecutive run of 11-mers, and all other 11-mers in the read are given x coordinates relative to this consecutive run. The y coordinate is read order as it appears in the input BAM file, and the color of each segment corresponds to the mapping position of its 11-mer.

### Strand-specific ccDNA sequencing

The same DNA sample was used as in “ccDNA sequencing by tagmentation”. DNA (500 ng) was treated with and without 100 U S1 Nuclease (Thermo Scientific) in 1× reaction buffer at 25°C for 2h before dilution with 50 mM Tris-HCl pH 7.5, extraction with phenol-chloroform-isoamylalcohol (25:24:1), and ethanol precipitation. Half of the precipitated DNA was used as input for strand-specific DNA libraries, prepared using a protocol adapted from Khan et al (16). Input DNA was supplemented with 1 fmol of 200 nt control ssDNA (ssd_spike) and incubated with 20 U terminal transferase (NEB) and 4 μM dATP in 1× reaction buffer (without CoCl_2_) at 37°C for 30 min then 70°C for 5 min. 2.5 pmol of primer ssExt_pT9_anchor was then annealed to the DNA, followed by addition of 1 mM dNTPs and 5 U Klenow Fragment (exo-, NEB). The reaction was incubated at 37°C for 30 min then 65°C for 5 min, and products were purified using the QIAquick PCR purification kit (Qiagen). Adapters ssd_adapt_top and ssd_adapt_bottom were annealed, and 100 fmol was ligated to 13% of the recovered DNA using Blunt/TA Ligase Master Mix (NEB). Ligation reactions were amplified with PhusionFlash PCR master mix and barcoded primers for 20 cycles. Amplicons larger than the 147 bp adapter dimer were purified by gel extraction and sequenced on a MiSeq in single-end mode (290 bp read 2). Reads were analyzed similarly to the tagmentation reads above, except that because ssDNA (or available 3′ OHs for terminal transferase extension) is not expected to be abundant in WT *E. coli*, except from the Ec86 retron, Okazaki fragments captured during DNA purification, and damaged DNA etc., we needed to normalize so as not to overestimate the abundance of ccDNA in uninfected cells. Indeed, the 200 nt ssd_spike added during all library preparations contributed to 80–90% of mapped reads in uninfected cells but only 9 – 12% in T5-infected cells, reflecting T5-induced genome degradation increasing the availability of genomic 3′ OHs, and also several nicks naturally present in the T5 genome (fig. S2). Therefore in Fig. 1F we weighted the y-coordinate of each displayed read by both the number of reads mapping to the ssd_spike, and by the culture volume equivalents necessary to provide the 500 ng input DNA to the S1 nuclease reactions.

### Small RNA sequencing

BL21-AI (Thermo Scientific) carrying pLG010 was grown in LB + Cm + 1 mM MgCl_2_ + 1 mM CaCl_2_ to an OD_600_ of 1.0. RNA was extracted using TRIzol (Thermo Scientific) and purified with a Direct-zol RNA MiniPrep kit (Zymo). The purified RNA was depleted of rRNA (RiboMinus, Thermo Scientific), treated with 20 units of T4 polynucleotide kinase (NEB), then 20 units of 5′ RNA polyphosphatase (Lucigen). After column purification, the RNA was prepared with the NEBNext Small RNA Library Prep (NEB) and sequenced on a MiSeq.

### ccDNA assays

Production of ccDNA was assayed by PCR or by real-time PCR. *E. coli* BL21-AI carrying pLG010 or variants, was grown to saturation at 37°C in LB containing Cm. 100 μL of each culture was diluted in 1 mL LB + Cm + 1 mM MgCl_2_ + 1 mM CaCl_2_ and grown to an OD_600_ of 0.75. 50 μL of each culture was mixed with T5 at an MOI of 10 in a 96-well PCR plate, and plates were incubated statically at 37°C for 80 min, or the indicated time. Samples of the infection were diluted 20-fold in water and heated at 95°C for 5 min before placing on ice. Boiled samples were diluted 10-fold into PCR reactions using either Phusion Flash (Thermo Scientific) for gel analysis, or SYBR Green PCR Master Mix (Applied Biosystems) for qPCR analysis. Several orthogonal pairs of primers were used, as indicated in Table S1, to avoid primers annealing to sequences mutated in particular assays. For gel analysis, PCR reactions were run for 20 cycles with 5 sec elongation times. For qPCR, PCR reactions were run for 40 cycles followed by melt curve analysis. qPCR reactions were thresholded as “not detected” if Cq > 30 or if the product did not have a detectable T_m_. qPCR reactions were normalized to a parallel qPCR on the same samples using primers specific to the expression plasmid that give an identically sized product (Table S1).

### Long-read sequencing of phage-infected cultures

1.5 mL of *E. coli* BL21-AI carrying pLG010 was grown in LB + Cm + 1 mM MgCl_2_ + 1 mM CaCl_2_ to an OD_600_ of 0.6 and infected with T5 at a MOI of 2. After 40 min at 37°C the culture was pelleted and total DNA was purified using the DNeasy Blood and Tissue Kit (Qiagen). Total DNA was directly input into the Nanopore gDNA ligation sequencing kit (SQK-LSK109, Oxford Nanopore) and sequenced on a MinION Flow Cell (Oxford Nanopore) for a total of 1,107,662 reads, with read polishing using Guppy.

### Bioinformatics

DRT2 orthologs analyzed in this study derive from the “group 9 UG2” systems previously classified (5). Group 9 loci were aligned and used to generate phylogenetic trees in Geneious Prime 2022.2.1 (52), and the 42 orthologs most closely related to *K. pneumoniae* DRT2 were extracted from the tree. ncRNA sequences were then extracted based on their alignment to the experimentally determined boundaries of the *K. pneumoniae* DRT2 ncRNA and realigned with Geneious and LocARNA (53). ccDNA sequences were inferred based on their alignment to the *K. pneumoniae* DRT2 template region and their bracketing by the conserved “ACA” motif. These regions were extracted, reverse complemented and concatenated in silico to produce theoretical ccDNA sequences. Open reading frames were identified in all ccDNA sequences and the corresponding amino acid sequences were realigned using MAFFT (54). A multiple sequence alignment of 5 concatenated repeats from the 42 orthologs was converted to a3m format and provided as the input for the ColabFold implementation of AlphaFold2 (21, 55) with settings --num-recycle 40 --num-models 5. To make AlphaFold3 models we supplied a five-repeat version of the *K. pneumoniae* DRT2 Neo protein to the web server (22). To calculate theoretical properties of the Neo ORF we used the ProtParam module of Biopython (56). For a “random” sequence control, we needed to control for biases in ccDNA codons that were due to selection at the DNA or RNA level. We generated randomized 120 bp ccDNA repeats that conserved the sequence and positions of the internal –10 element (fixed as TATAAT), –35 element (fixed as TTGACA), repeat junction (fixed as TGTTA) and ribosome binding site (fixed as GAG). The gaps between these sequences were filled with randomly selected codons (all 61 coding codons with an equal chance of being selected). The randomly generated sequences were then concatemerized into 20-repeat units before translation and property calculation.

### ccDNA transcription assays

*E. coli* BL21-AI carrying pLG010 or variants was grown to saturation at 37°C in LB containing Cm. 150 μL of each culture was diluted in 3 mL LB + Cm + 1 mM MgCl_2_ + 1 mM CaCl_2_ and grown to an OD_600_ of 1. Cultures (1 mL aliquots) were mixed with T5, T2, or T4 at an MOI of 10 and incubated at 37°C for 30 min before pelleting. RNA was purified from the pellets using Tri-reagent (Zymo) and the Direct-zol RNA MiniPrep kit (Zymo) with on-column DNase I digestion. 250 ng of RNA was annealed to 1 pmol of ccDNA primer 4 reverse (Table S1) and reverse transcribed with SuperScript IV (Invitrogen). Control reactions were processed identically but omitting the SuperScript IV enzyme. The reverse transcription reactions were diluted 10-fold in a Phusion Flash PCR reaction mixture with ccDNA primers 4 (Table S1) and amplified with 20 cycles and 5 sec elongation times before analysis by agarose gel electrophoresis and SYBR Gold staining.

### Transcriptional reporter assays

Fluorescent reporters were created by cloning 120 bp ccDNA repeat junctions in front of the start codon of GFPmut2 in high-copy number pUC plasmids and transforming into NEB 5-alpha cells, giving plasmids pMW322, pMW323, pMW324, and pMW325 (Table S1). Overnight cultures were diluted 20-fold in LB + ampicillin and grown in a 96-well plate in a Synergy Neo2 plate reader at 37°C (BioTek). GFP fluorescence (488/528 nm filter) and OD_600_ were measured every 5 min. To compare the cultures, we took the GFP fluorescence values at the timepoint where each culture crossed OD_600_ of 0.5 and subtracted the background from a media control. *E. coli* displays autofluorescence so we included a control culture with no GFP plasmid to act as a background control.

### Growth assays

For liquid culture experiments, 100 μL cultures in LB were grown in clear-bottom 96-well plates in a Synergy Neo2 plate reader at 37°C (BioTek). OD_600_ values were measured every 5 min. For plate assays to assess Neo toxicity, overnight cultures in LB + 0.2% glucose + ampicillin were serially ten-fold diluted in LB. 3 μL of each dilution was spotted onto LB agar plates containing 0.2% glucose or the indicated concentration of arabinose, allowed to dry, then inverted and incubated overnight at 37°C. Plates were imaged with a white backlight.

### DRT2 RNP purification

The T7-inducible DRT2 operon with a C-terminal twin-StrepII tag expression plasmid (pMW116) was transformed into *E. coli* BL21(DE3) (NEB) and grown at 37°C in Terrific Broth supplemented with 25 mM disodium hydrogen phosphate, 25 mM potassium dihydrogen phosphate, 50 mM ammonium chloride, 5 mM sodium sulfate, 0.5% (w/v) glycerol, 0.2% (w/v) α-lactose monohydrate, 0.05% (w/v) glucose, 2 mM magnesium chloride (TB-based autoinduction media), and 50 μg/L ampicillin. The temperature was reduced to 18°C during mid-log phase, and cells were grown for another 16 hr. Cells were harvested, frozen, and suspended in Lysis Buffer (50 mM Tris-HCl pH 7.4, 500 mM NaCl, 5% glycerol, 5 mM beta-mercaptoethanol) supplemented with EDTA-free cOmplete protease inhibitor (Roche). Cells were lysed using a LM20 microfluidizer device (Microfluidics), and cleared lysate was bound to Strep-Tactin Superflow Plus resin (Qiagen). Resin was washed first with lysis buffer and then with DRT2 Buffer (10 mM HEPES-KOH pH 7.9, 250 mM potassium acetate, 2.5 mM magnesium acetate, 0.5 mM TCEP) before elution with DRT2 Buffer supplemented with 5 mM desthiobiotin. Fractions containing protein and RNA were concentrated in an Amicon Ultra-4 centrifugal concentrator (30000 MWCO; Millipore) until OD260 nm = 92. For the purification intended for cryo-EM determination in the presence of dNTPs, DRT2 Buffer was substituted with 20 mM HEPES-KOH pH 7.9, 150 mM potassium acetate, 2 mM magnesium acetate and the sample was concentrated to OD260 nm = 61.

### BoMoC (truncated R2Bm RT) and DBR1 purification

pMW342 for expressing BoMoC derives from pET-His-MBP-SUMO-R2Bm-SII deltaN (57) with the D996A mutation introduced by Gibson Assembly, the resulting plasmid is similar to the previously described BoMoC construct (24). pMW340 for expressing yeast DBR1 is derived from the same plasmid, but with the sequence between the T7 promoter and SII tag replaced with DBR1 amplified from yeast genomic DNA.

*E. coli* BL21(DE3) containing pMW342 or pMW340 was grown and induced as described for the DRT2 RNP. BoMoC was purified as described (57) with some modifications. Cells were suspended in R2 Lysis Buffer (50 mM Tris-HCl pH 7.4, 1 M NaCl, 10% glycerol, 5 mM beta-mercaptoethanol, 1 mM MgCl_2_, 0.5 mM PMSF) and lysed with an LM20 microfluidizer device (Microfluidics), and cleared lysate was bound to Strep-Tactin Superflow Plus resin (Qiagen). The resin was washed with R2 Wash Buffer (20 mM HEPES-KOH pH 7.9, 1M KCl, 10% glycerol, 1 mM DTT), then with R2 Storage Buffer (20 mM HEPES-KOH pH 7.9, 800 mM KCl, 10% glycerol, 1 mM DTT) before elution with R2 Storage Buffer supplemented with 5 mM desthiobiotin. Protein-containing fractions were pooled and concentrated in an Amicon Ultra-4 centrifugal concentrator (30000 MWCO; Millipore) until OD280 nm = 7.1 and OD260 nm = 4.3, or 36 μM. A 10 μM working stock was prepared by dilution in storage buffer containing 69% glycerol (to final concentration 50% glycerol) and stored at −20°C; the rest of the protein was flash frozen in liquid nitrogen and stored at −80°C.

DBR1 was purified identically except that the lysis buffer was 50 mM Tris-HCl pH 7.4, 250 mM NaCl, 10% glycerol, 5 mM beta-mercaptoethanol, 0.5 mM PMSF, the Wash Buffer was 20 mM HEPES-KOH pH 7.9, 500 mM NaCl, 10% glycerol, 1 mM DTT, and the Storage Buffer was 20 mM HEPES-KOH pH 7.9, 250 mM NaCl, 10% glycerol, 1 mM DTT.

### DRT2-associated nucleic acid purification for OTTR sequencing

Primer-binding site libraries were generated by PCR amplification of pMW116 with primers mutate_PBS_R and PBS_N1–9_R, Gibson assembly, and pooling all colonies before plasmid purification. To purify nucleic acids associated with the DRT2 RT, *E. coli* BL21(DE3) containing pMW116, pMW343, or primer-binding site libraries was grown as described for “DRT RNP purification.” A 0.1 – 0.2 g piece of frozen cell pellet was suspended in 1 mL Lysis Buffer supplemented with 0.5 mM PMSF and sonicated on ice for 30 sec. Cell debris was pelleted by centrifugation at 21000*g* for 10min, and the supernatant was bound to Strep-Tactin Superflow Plus resin. After washing 5 times with Lysis Buffer, the resin was extracted with 25:24:1 phenol/chloroform/isoamyl alcohol, ethanol precipitated, and the pellet dissolved in water.

### Ordered Two-Template Relay (OTTR) Sequencing

Nucleic acids associated with DRT2 were sequenced by Ordered Two-Template Relay as previously described (24), with some modifications. 10 μL of 100 ng/μL input nucleic acids in water were heated to 95°C for 2 min and immediately placed on ice. They were then mixed with 20 mM Tris-HCl pH 7.5, 200 mM KCl, 2 mM DTT, 5% PEG-8000, 2 mM ammonium sulfate, 0.7 mM sodium acetate, 2 mM MnCl_2,_ 250 μM ddATP, and 0.7 μM BoMoC. One reaction with nucleic acids from WT DRT2 RNP additionally contained 14 nM DBR1. After incubation at 30°C for 90 min, 5 mM MgCl_2_ and 0.5 U of shrimp alkaline phosphatase (NEB) were added, incubated at 37°C for 15 min, before addition of 5 mM EGTA and incubation at 65°C for 5 min. Then, 0.5 mM MgCl_2_, 85 mM KCl, 200 μM GTP, 40 μM dTTP, 40 μM dCTP, 40 μM dATP, 90 nM RNA-DNA primer duplex (OTTR_c5_primer + OTTR_c5_RNA), 180 nM adapter template (OTTR_c3_AT), and 0.5 μM BoMoC were added. The mixture was incubated at 37°C for 20 min then 65°C for 5 min. 1 μL of the reaction was then directly used as template in a 50 μL PCR with barcoded primers and PhusionFlash polymerase for 20 cycles and 30 seconds per elongation step. Adapter dimers were removed by pooling PCR products and purification with 0.6 volumes of SPRIselect beads (Beckman Coulter). Libraries were sequenced on a MiSeq in single-end mode (290 bp read 2).

### In vitro DRT2 assays

DRT2 RNP (stored at −80°C) was diluted to OD260 nm = 1 in 20 mM HEPES-KOH pH 7.9, 150 mM potassium chloride, 10 mM magnesium acetate, and the reaction was started by addition of 0.25 mM each dNTP (or the indicated concentration). Reactions were incubated at 37°C for 30 min and stopped by addition of Proteinase K. Reaction products were digested with RNase A and visualized by agarose gel electrophoresis (2% E-Gel, Invitrogen) or denaturing PAGE (10% dPAGE, Invitrogen) and SYBR Gold staining. For analysis by OTTR sequencing, a 100 μL reaction with 16 μM of each dNTP was stopped by addition of 10 U shrimp alkaline phosphatase (NEB), incubation at 37°C for 15 min, followed by addition of 55 μL 1M NaOH and incubation at 70°C for 15 min, addition of 55 μL 1M HCl, dilution to 400 μL with 100 mM Tris-HCl pH 7.5 and phenol/chloroform/isoamyl alcohol (25:24:1) extraction.

### Long-read sequencing of in vitro ccDNA products

We designed a Nanopore library preparation protocol that could sequence both ssDNA and dsDNA. DNA from an 80 μL in vitro DRT2 reaction that used 0.25 mM each dNTP was extracted with phenol/chloroform/isoamyl alcohol and precipitated with ethanol. The precipitate was dissolved, treated with RNase A, and ligated to a 5′ pre-adenylated ssDNA adaptor ( /5rApp/CTGTCTCTTATACACATCTCCGAGCCCACGAGAC/3SpC3/) using thermostable 5′ App DNA/RNA ligase (NEB). The reaction was treated with Proteinase K and products were purified with RNAclean XP beads. 0.5 uM of a ssDNA oligo complementary to the adaptor was added along with Phusion Flash master mix, and 8 cycles of polymerization were performed to allow second strand synthesis for any ssDNA in the reaction. Products were then purified and treated with the NEBNext Ultra II End Prep Repair/dA-tailing kit (NEB), purified with AMPure XP beads (Beckman Coulter) and ligated to the Oxford Nanopore Adaptor using the Ligation Sequencing Kit V14 (Oxford Nanopore). The library was loaded on a MinION Flow Cell (Oxford Nanopore) and sequenced for a total of 1,606,094 reads, with read polishing using Guppy. To quantify repeats per read, reads were analyzed with RepeatMasker. To quantify template jumps, reads were split into consecutive 11-mers (the minimum length for unique hashing) and each 11-mer was assigned to its corresponding position and strand within the ncRNA. Jumps were quantified as non-adjacent mapping of adjacent 11-mers.

### Cryo-EM sample preparation and data collection

Freshly purified DRT2 RNP (OD260 nm = 92 or 18) was applied to a glow-discharged (60 s at 25 mA) Cu300 R1.2/1.3 holey carbon grid (Quantifoil) mounted in the chamber of a Vitrobot Mark IV (Thermo Fisher Scientific) maintained at 12°C and 100% humidity. Grids were blotted using Ø55 grade 595 filter paper (Ted Pella) for 4 s and plunged into liquid ethane. For the structure containing dNTPs, DRT2 RNP at OD260 nm = 61 was mixed with 0.2 mM each dNTP and incubated at 23°C for 150 sec before plunge freezing. Cryo-EM data were collected using the Thermo Scientific Titan Krios G3i cryo TEM at MIT.nano using a K3 direct detector (Gatan) operated in super-resolution mode with 2-fold binning, and an energy filter with slit width of 20 eV. Micrographs were collected automatically using EPU in AFIS mode at 130,000× magnification with a real pixel size of 0.663 Å.

For the resting (no dNTP) structure, two grids were imaged, one with a higher concentration of DRT2 RNP applied. 14,220 movies for grid 1 were collected and 13,991 movies for grid 2. Both sets of micrographs had an exposure time of 0.9 s, fractionated into 40 frames and a flux of 19.9 e^−^/pix/s giving a total fluence per micrograph of 40.7 e^−^/Å^2^. For the dNTP structure, one grid was imaged. 18,453 movies were collected, using an exposure time of 0.87 s, fractionated into 40 frames and a flux of 20.35 e^−^/pix/s giving a total fluence per micrograph of 40.3 e^−^/Å^2^.

### Cryo-EM data processing

All cryo-EM data were processed using RELION-4.0 (58). Movies were corrected for motion using the RELION implementation of MotionCor2, with 6×4 patches and dose-weighting. CTF parameters were estimated using CTFFIND-4.1 (59).

For the resting structure (no dNTPs), each grid was initially processed separately. Particle picking was done using Topaz with the general model (60) and a 110 Å particle diameter, yielding 2,510,771 particles (grid 1) or 2,117,653 particles (grid 2). These were extracted with a box size of 288 pixels and binned during extraction to 72 pixels, 2.652 Å/pix. An initial model was generated from 81,061 particles selected from grid 1 by two rounds of 2D classification during on-the-fly processing. One round of 3D classification, using this initial model with a 20 Å low-pass filter, --iter 25 --tau2_fudge 6 --K 4 --fast_subsets --maxsig 500, was used to select 637,675 (grid 1) or 505,156 (grid 2) promising particles, which were re-extracted with a 144 pixel box, 1.326 Å/pix, and refined to 3.54 Å (grid 1) or 3.47 Å (grid 2) resolution. 3D classification without alignment, --iter 25 --tau2_fudge 8 --K 4, yielded 123,816 particles from grid 1 and 106,185 from grid 2 that had more well-defined features. These particles from the two grids were merged, defocus parameters were refined, and after 3D refinement gave a 3.13 Å resolution map. Particles from each grid were subjected to Bayesian polishing separately, using the trained parameters --s_vel 1.026 --s_div 8055 --s_acc 2.04 for grid 1 and --s_vel 1.202 --s_div 8607 --s_acc 1.413 for grid 2. The polished particles were extracted using a 320 pixel box and binned to 240 pixels, 0.884 Å/pix. A final refinement after merging the polished particles produced an isotropic map at 2.91 Å resolution with features consistent with the estimated resolution.

For the dNTP structure, a Topaz particle picking model was trained by picking a random subset of 300 micrographs with the Topaz general model and using two rounds of 3D classification to select 22,514 high quality particles, which were used for training. With the trained model, 3,313,207 particles were picked and extracted with a 288 pixel box and binned to 72 pixels, 2.652 Å/pix. 3D classification using the resting structure as a reference, low-pass filtered to 20 Å, --iter 25 --tau2_fudge 4 --fast_subsets --K 4, allowed selection of a 1,218,945 particle subset which was re-extracted with a 144 pixel box, 1.326 Å/pix, and refined to 3.35 Å resolution. These particles contained a mixture of states, predominantly resembling the resting structure. To identify particles that were actively reverse transcribing, we performed 3D classification without alignment with a mask that would exclude the flexible template region, --iter 36 --K 8 --tau2_fudge 16. 136,601 particles (11%) showed a shifted 3′ terminus indicative of elongation. These were reextracted using a 320 pixel box, binned to 240 pixels (0.884 Å/pix), refined to 3.26 Å resolution, and improved by per-particle defocus refinement and Bayesian polishing (trained parameters --s_vel 1.163 --s_div 12030 --s_acc 2.28) to produce a final map at 3.17 Å resolution. All resolutions are reported using the gold-standard Fourier Shell Correlation with 0.143 cutoff.

### Cryo-EM model building

An AlphaFold2 model of the DRT2 RT was docked into the resting cryo-EM density and fitted using ISOLDE (61). The ncRNA was built de novo and the protein side chains adjusted manually using Coot (62). The model was refined first using ISOLDE, then with Phenix real_space_refine (63), just performing one macro-cycle. For the dNTP structure, the resting structure was fit into the cryo-EM density using ISOLDE. The template region outside of the template/primer duplex was deleted. The sequence of the nucleotides of the template/primer duplex and incoming dNTP cannot be read from the cryo-EM density, consistent with an elongating state with each particle being a different distance along the template. We arbitrarily modelled the incoming dNTP as dTTP. The model was then refined with Phenix. Cryo-EM data statistics and model statistics can be found in Table S2.

## Supplementary Material

supplemental material

Table S1

## Data Availability

The cryo-EM maps for the DRT2 RNP have been deposited in the Electron Microscopy Data Bank with accession codes EMD-45085 (resting state) and EMD-45086 (elongating state). The coordinates for the atomic modes have been deposited in the Protein Data Bank under accession codes 9C0I and 9C0J. Sequencing data have been deposited in the NCBI Sequence Read Archive under BioProject ID PRJNA1144948. Scripts for data analysis are available at https://github.com/maxewilkinson/DRT2-analysis (https://doi.org/10.5281/zenodo.13257156) (67).
