# Supplementary material for: Phage-triggered reverse transcription assembles a toxic repetitive gene from a non-coding RNA
Source: Science. Author manuscript; Available in PMC 2025 Apr 29. (PMC12039810; doi:10.1126/science.adq3977)
Supplement: supplemental material [file NIHMS2071915-supplement-supplemental_material.pdf]

Science
AAAS

# Supplementary Materials for

## Phage-triggered reverse transcription assembles a toxic repetitive gene from a non-coding RNA

Max E. Wilkinson, David Li, Alex Gao, Rhiannon K. Macrae, Feng Zhang

Corresponding author: zhang@broadinstitute.org

**The PDF file includes:**

Figs. S1 to S9
Tables S1 to S2

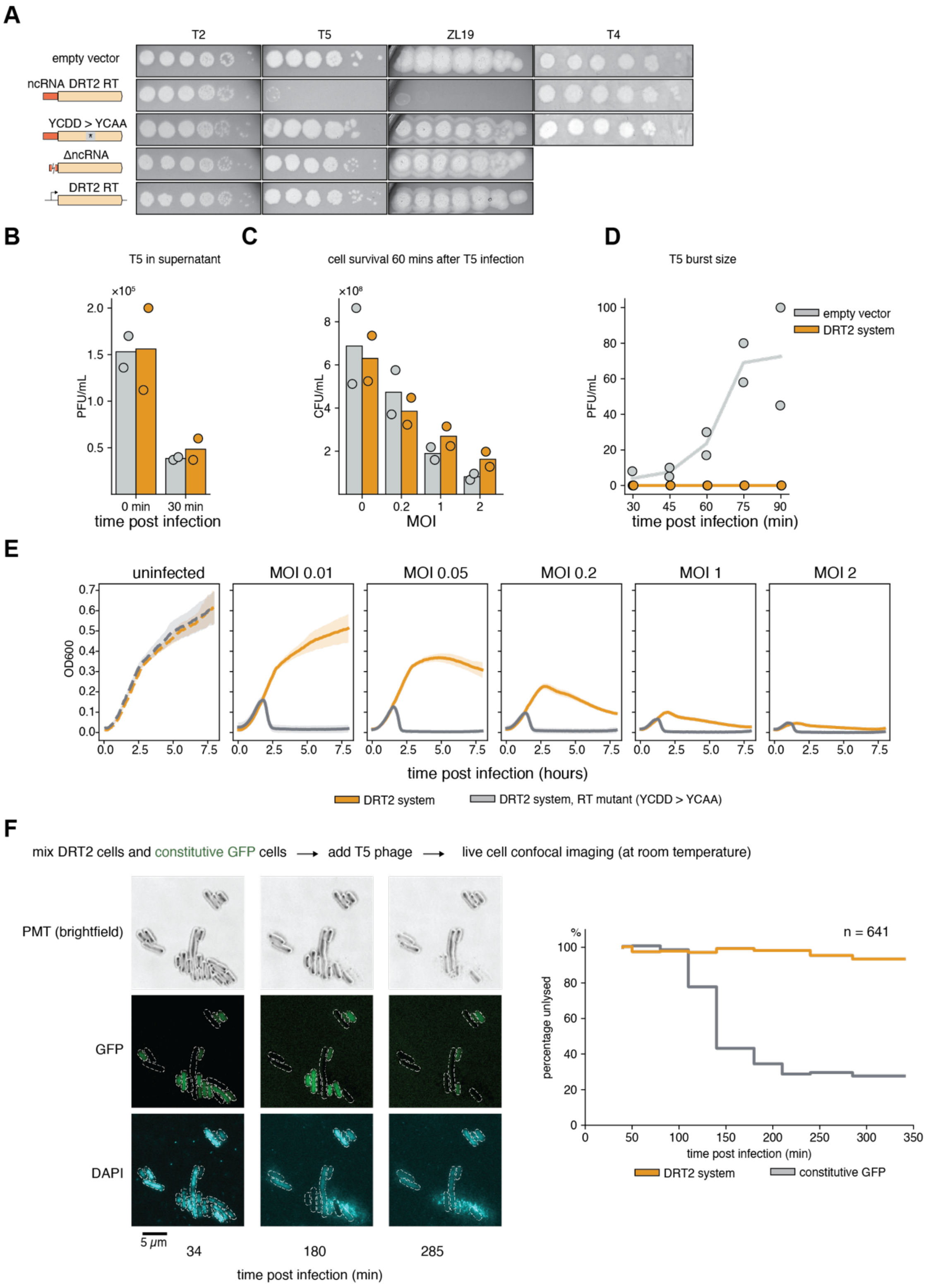


**Fig. S1.**

Characterization of the phage defense activity of DRT2. **A)** Photographs of plaque assays on soft-agar lawns of BL21-AI *E. coli* carrying an empty vector or DRT2 defense system variants.

Ten-fold dilution series of the indicated phage were spotted on the lawns. YCDD>YCAA is a reverse-transcriptase active site mutant. The ΔncRNA and constitutive promoter-driven RT constructs (pLG616 and pLG554) were previously described (*4*). **B)** Phage adsorption assay. Adsorption of T5 phage to BL21-AI *E. coli* was assessed with and without the DRT2 defense system (empty vector control). **C)** Cell-survival assay. Survival of BL21-AI *E. coli* with and without the DRT2 defense system was assessed by plating and counting colony-forming units (CFUs) after 60 mins of infection with the indicated multiplicity of infection (MOI) of T5 phage. **D)** Amount of new T5 production in the supernatant of infected cultures was assessed in of BL21-AI *E. coli* with and without the DRT2 defense system (two replicates for each time point and condition). **E)** Growth curves of BL21-AI *E. coli* expressing the DRT2 defense system or the YCDD>YCAA active-site mutant at differing MOIs of T5 phage. Lines show the means and shading shows plus and minus the standard error of four replicates. **F)** Live-cell imaging of BL21-AI *E. coli* expressing DRT2 or constitutive GFP. Left: an example region from the full field of view at three timepoints. Dashed lines, cells segmented using Omnipose. Right: survival curve from the full field of view which contained 641 cells at the first time point (341 GFP positive cells, 300 GFP negative cells).

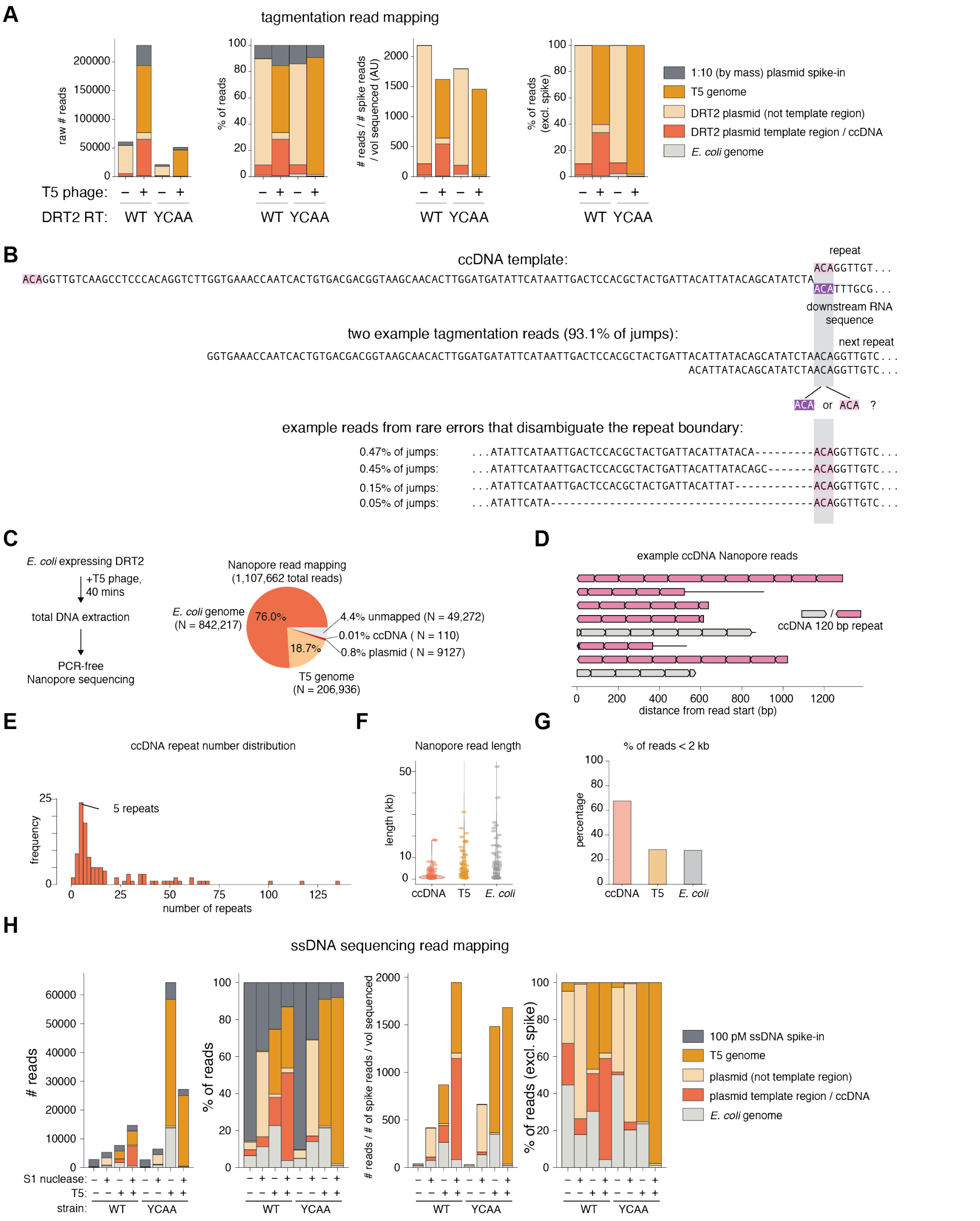


**Fig. S2.**

Characterization of ccDNA produced in vivo. **A)** Related to **Fig. 1B**. Distribution of tagmentation read mapping to different reference sequences in absolute read numbers (left) and

as percentage of total reads (right). **B)** Example ccDNA reads from Tn5 sequencing of phage-infected *E. coli* expressing DRT2. The top sequence shows a 120 bp full template region (in the ncRNA/plus-strand sense) with an "ACA" sequence on the left, and either the first 9 bp of the next repeat as in ccDNA, or the first 9 bp of the downstream ncRNA sequence. Both start with "ACA," making it ambiguous precisely where the jump occurs (i.e. whether the rightmost or leftmost "ACA" is part of the repeat template). Four rare examples of incorrect template jumps are shown that together suggest the leftmost "ACA" is the repeat template, not the rightmost "ACA". **C)** The proportion of Nanopore reads mapping to the host genome, T5 phage, DRT2 expression plasmid, or to the ccDNA. **D)** Example Nanopore reads mapping to the ccDNA. Reads were annotated using RepeatMasker and contain tandem repeats of the DRT2 ncRNA template region. **E)** Frequency distribution of number of template repeats found per Nanopore read, as determined by RepeatMasker annotation. **F)** The distribution of Nanopore read lengths for reads mapping to the host genome, T5 phage, or ccDNA. **G)** Percentage of Nanopore reads, mapped to the host genome, T5 phage, or ccDNA that are shorter than 2000 bp. **F** and **G** suggest that the size of the ccDNA estimated from Nanopore read lengths is shorter than the average read length in the library. **H)** Related to **Fig. 1F**. Distribution of strand-specific ssDNA sequencing read mapping to different reference sequences in absolute read numbers and as percentage of total reads, with and without normalization to the number of reads mapping to the 100 pM ssDNA spike.

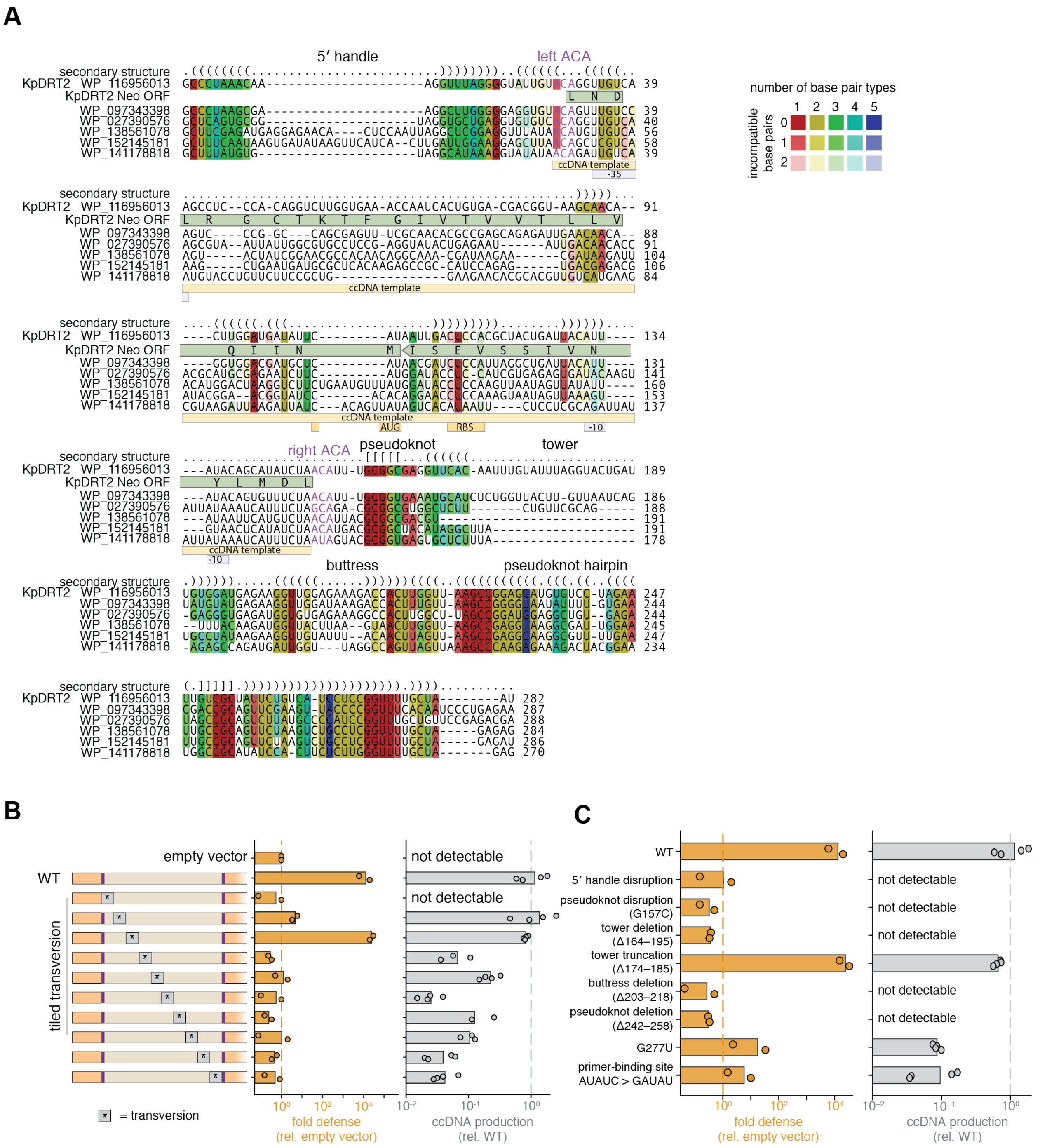


**Fig. S3.**

Multiple sequence alignment of the DRT2 ncRNA. **A)** Six representatives of close relatives of the *K. pneumoniae* DRT2 (KpDRT2) ncRNA were aligned using LocARNA (*53*). The alignment and predicted secondary structure were adjusted to match the cryo-EM structure, including notating the pseudoknot and removing most predicted pairs within the template region. Bases are colored according to their degree of covariation within the indicated secondary structures. The open reading frame hidden within the template region is indicated for KpDRT2 with a backwards green arrow, starting at the predicted start codon and permuting back to before the start codon to form one Neo repeat unit. **B)** Mutation of the DRT2 template region. 10 non-overlapping 12-bp

tiled transversions were made and defense against T5 phage was determined as the fold-reduction in plaque forming units (PFUs) (yellow plot). ccDNA production after T5 infection was determined by qPCR (gray plot). Individual measurements are shown as dots. **C)** T5 defense (fold reduction in PFUs) and T5-induced ccDNA production (qPCR) for mutants in the ncRNA, compared to a wild-type control. 5′ handle disruption: mutation of $_{14}$GUUUAGGG$_{21}$ to $_{14}$CCCUAAAC$_{21}$.

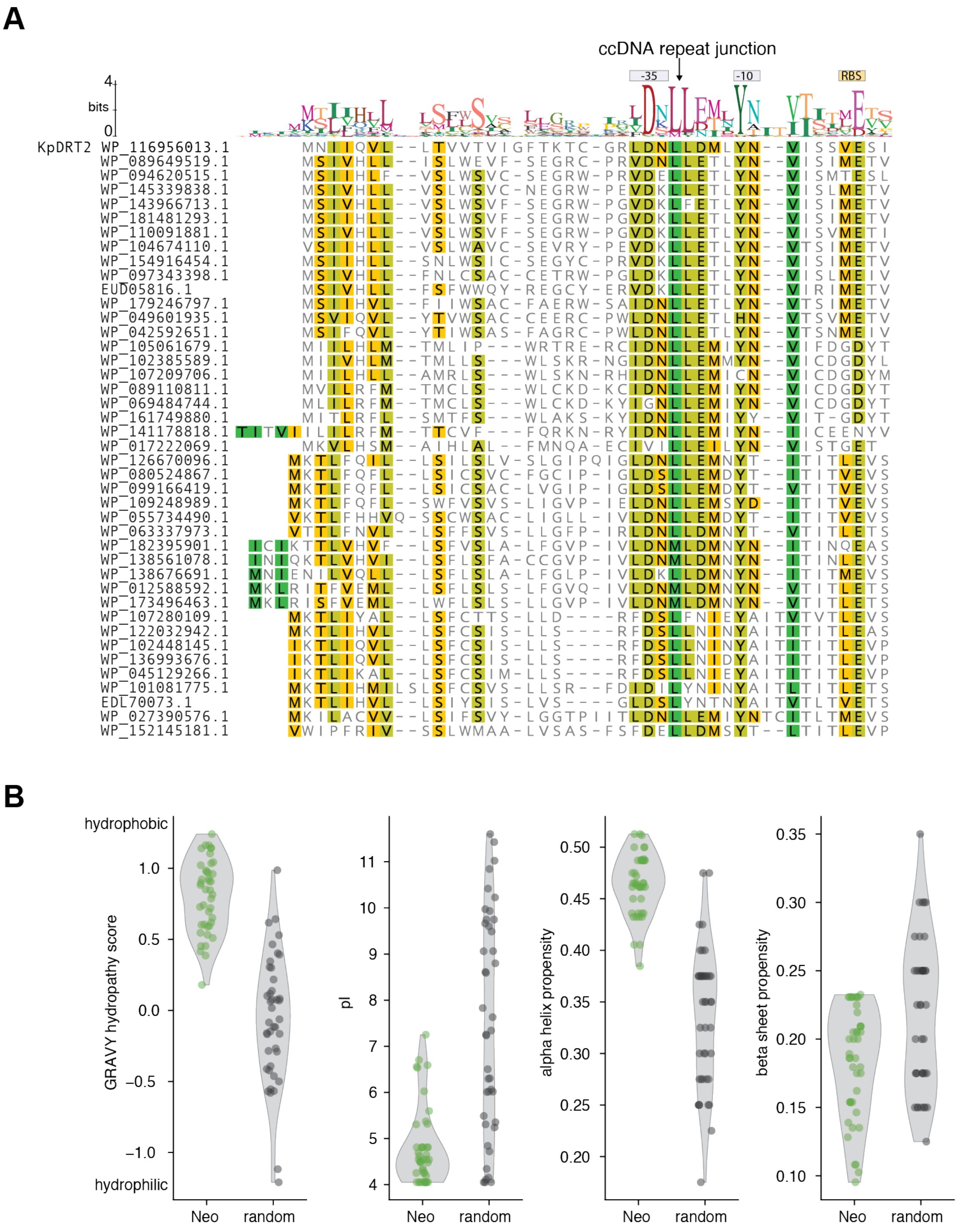


**Fig. S4.**

Multiple sequence alignment and properties of the Neo ORF. **A)** Translated sequences of a single repeat unit of the Neo ORF were predicted for 42 orthologs of the KpDRT2 system. The repeats are shown aligned to the predicted start codon for KpDRT2. Most repeats also have a start codon in this position or nearby, but some do not, suggesting some orthologs might initiate translation at a different position within the repeat. Sequences highly conserved at the nucleotide level, like the

promoter elements, are indicated, since the nucleotide biases of these elements mostly accounts for the amino acid conservation at these positions. **B)** Predicted sequence properties of 20-repeat version of these 42 Neo ORF orthologs. Properties were calculated using Biopython (*56*) and distributions of properties were plotted. The randomized ORFs conserve the promoter and other such nucleotide-level properties of the Neo ORFs, see methods for details.

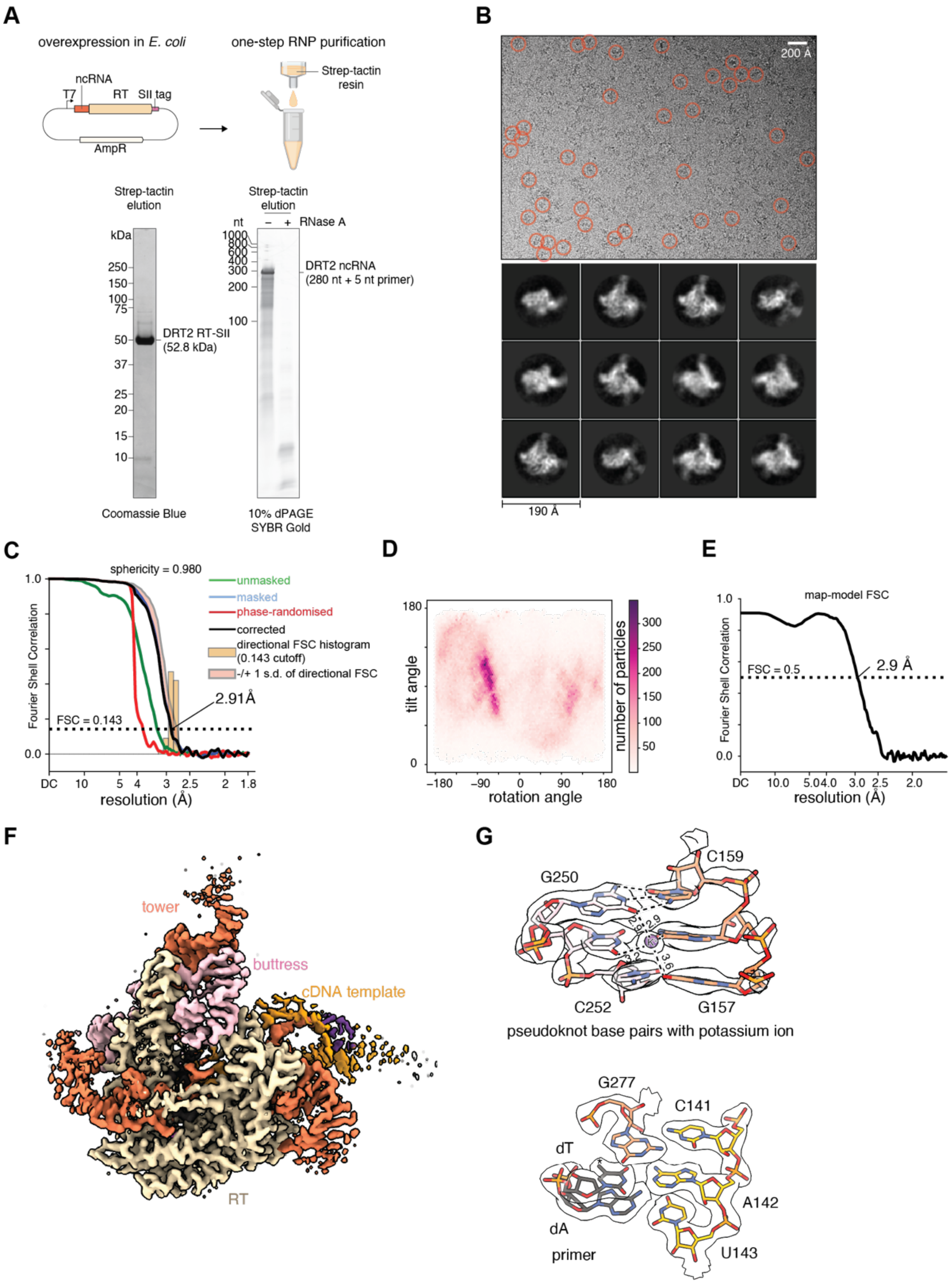


## Fig. S5.

Purification and cryo-EM analysis of the DRT2 ribonucleoprotein complex. **A)** RNP purification. Schematic of the purification strategy used. The peak elution fraction is shown analyzed by SDS-PAGE and Coomassie Blue staining, showing a major band corresponding to the DRT2 reverse transcriptase. The same fraction was extracted with phenol-chloroform-isoamyl alcohol and run on a 10% denaturing PAGE gel stained with SYBR Gold. The fraction contains a <300 nt nucleic acid sensitive to RNase treatment, consistent with being the 285 nt

DRT2 ncRNA+primer. **B)** Example cryo-EM micrograph of the DRT2 RNP. Circled particles are in the final reconstruction. Selected 2D class averages are shown below. The circular mask is 120 Å in diameter. **C)** Gold-standard Fourier Shell Correlation (FSC) curves and 3DFSC (*65*) plots for the final reconstruction of the DRT2 RNP in the resting state. **D)** Orientation distribution heat map for the DRT2 RNP reconstruction. **E)** Map-model FSC curve for the DRT2 RNP. **F)** Cryo-EM density for the DRT2 RNP in the resting state. **G)** Example cryo-EM densities for the DRT2 RNP (resting state). A potassium ion in the pseudoknot is assigned based on the 2.9–3.2 Å coordination distances and octahedral geometry (as well as potassium being present in the purification buffer). The cryo-EM density of the primer/template duplex is consistent with the primer being DNA, based on the 5-methyl group on the thymidine base (asterisk).

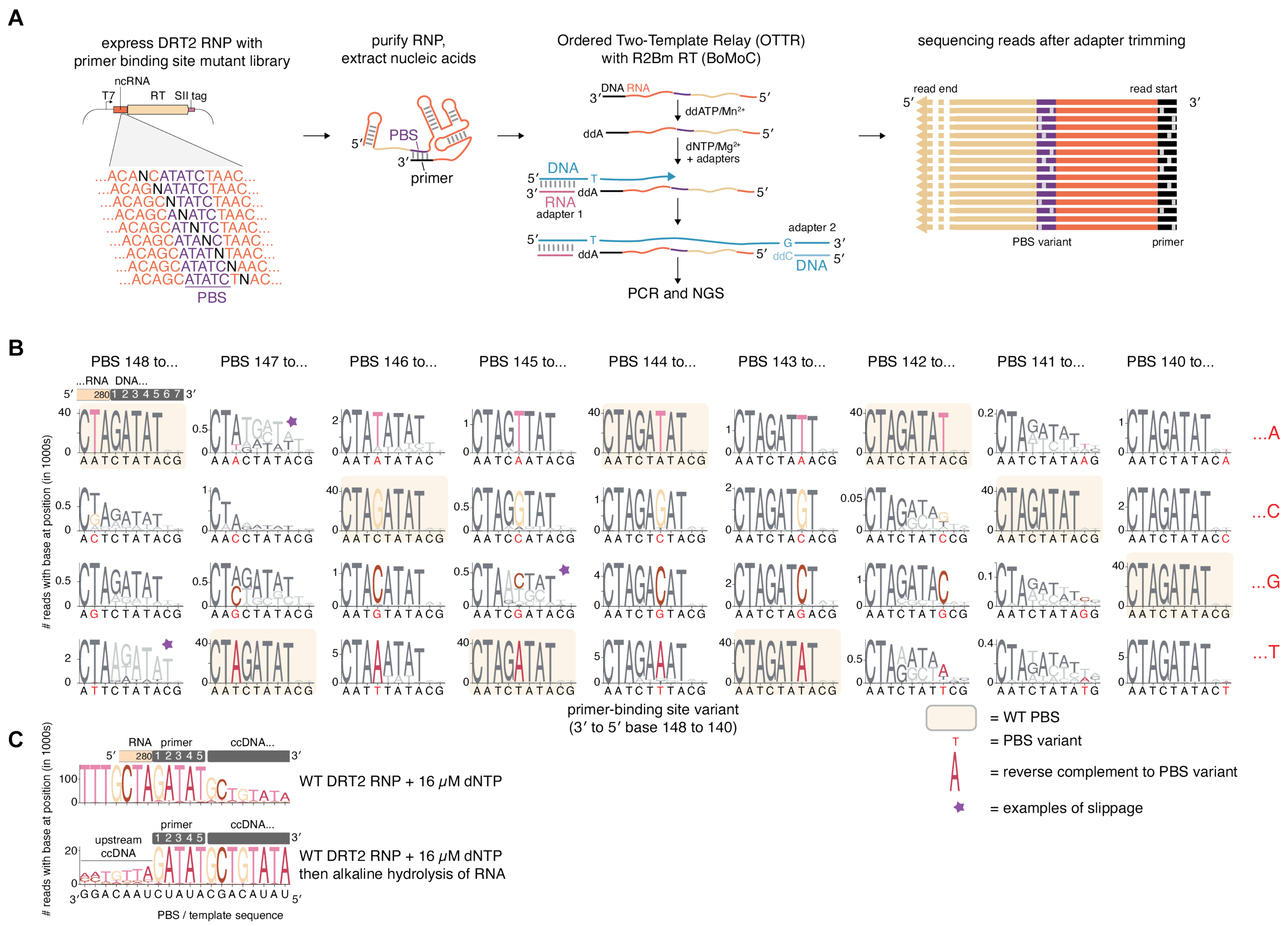
A
express DRT2 RNP with
primer binding site mutant library
ncRNA
T7
RT
SII tag
...ACANCATATCTAAC...
...ACAGNATATCTAAC...
...ACAGCNTATCTAAC...
...ACAGCANATCTAAC...
...ACAGCATNTCTAAC...
...ACAGCATANCTAAC...
...ACAGCATATNTAAC...
...ACAGCATATCNAAC...
...ACAGCATATCTNAC...
PBS
purify RNP,
extract nucleic acids
PBS
primer
Ordered Two-Template Relay (OTTR)
with R2Bm RT (BoMoC)
DNA RNA
ddATP/Mn²⁺
ddA
dNTP/Mg²⁺
+ adapters
DNA
RNA
adapter 1
adapter 2
PCR and NGS
sequencing reads after adapter trimming
read end
read start
PBS variant
primer
B
PBS 148 to...
PBS 147 to...
PBS 146 to...
PBS 145 to...
PBS 144 to...
PBS 143 to...
PBS 142 to...
PBS 141 to...
PBS 140 to...
...RNA DNA...
# reads with base at position (in 1000s)
...A
...C
...G
...T
primer-binding site variant
(3′ to 5′ base 148 to 140)
= WT PBS
= PBS variant
= reverse complement to PBS variant
= examples of slippage
C
RNA primer
ccDNA...
WT DRT2 RNP + 16 μM dNTP
upstream
ccDNA
WT DRT2 RNP + 16 μM dNTP
then alkaline hydrolysis of RNA
PBS / template sequence

**Fig. S6.**

Effects of all primer-binding site mutations on primer synthesis. **A)** Schematic of Ordered Two-Template Relay (OTTR) sequencing to simultaneously sequence DRT2 ncRNA primer-binding site variants and the resultant primer sequences (*24*). **B)** Sequence logos (*66*) for the final three nucleotides of the DRT2 ncRNA ($CUA_{280}$) and the primer sequences, for each primer-binding site variant. Each column is a different mutated position, each row is the base mutated to. The logo for the WT primer-binding site is therefore reproduced once per column (highlighted background). The x-axis labels for each logo shows the primer-binding sequence reversed, aligned according to its base-pairing with the primer in the DRT2 RNP cryo-EM structure. Dark grey highlights the WT sequence, colored bases are complementary to the mutation introduced. Most mutations result in complementary changes to the primer, supporting the primer-binding site templating synthesis of the primer. Position 140 has no effect on the primer sequence, as it does not base pair to any primer bases. Any change to position 141 (which pairs with G277), or U148 to C or A, severely inhibits primer synthesis or leads to heterogeneous primers. Interestingly A149>U leads primer synthesis being templated one base earlier in the primer-binding site, resulting in a 1 nt longer primer. **C**) OTTR sequencing of nucleic acids from WT DRT2 RNPs incubated with 16 $\mu$M dNTPs. This concentration of nucleotides mainly leads to accumulation of shorter products amenable to sequencing (**Fig. 5A**). Reads mapping to the PBS minus strand or to the ncRNA 3′ end plus strand were aligned and used to calculate sequence logos. This shows that the covalently linked primer is indeed extended during ccDNA synthesis. The reaction products were purified and treated first with alkaline phosphatase then 333 mM sodium hydroxide at 70°C for 15 min for acid neutralization, purification and OTTR sequencing. The "GATAT" part of the primer is resistant to alkaline hydrolysis, supporting it being DNA and the upstream sequences being RNA. **B** indicates that PBS U147G can weakly template a CGATAT primer, suggesting some 3′ end heterogeneity on the ncRNA before primer synthesis may occasionally lead to a 6 nt DNA primer.

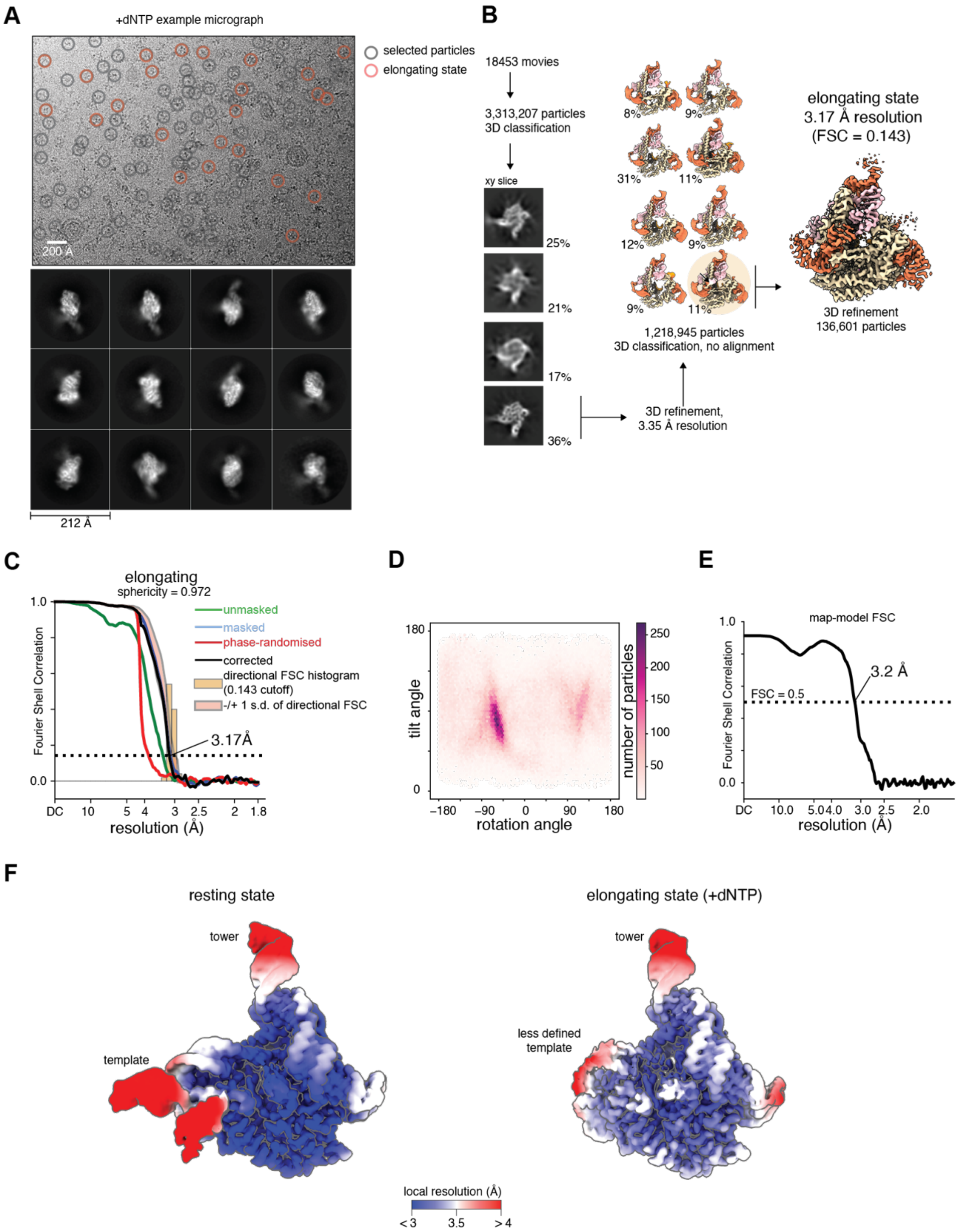


**Fig. S7.**

Cryo-EM analysis of the DRT2 ribonucleoprotein complex in the elongating state. **A)** Example cryo-EM micrograph of the DRT2 RNP + dNTPs. Particles circled with black were selected for classification, particles circled in red are in the final reconstruction of the elongating state. Selected 2D class averages for the elongating state are shown below. **B)** Cryo-EM processing scheme showing how the elongating state was selected. **C)** Gold-standard Fourier Shell

Correlation (FSC) curves and 3DFSC (*65*) plots for the final reconstruction of the DRT2 RNP in the elongating state. **D)** Orientation distribution heat map for the elongating DRT2 RNP reconstruction. **E)** Map-model FSC curve for the elongating DRT2 RNP. **F)** Final maps for the resting and elongating states of the DRT2 RNP, filtered and colored by local resolution.

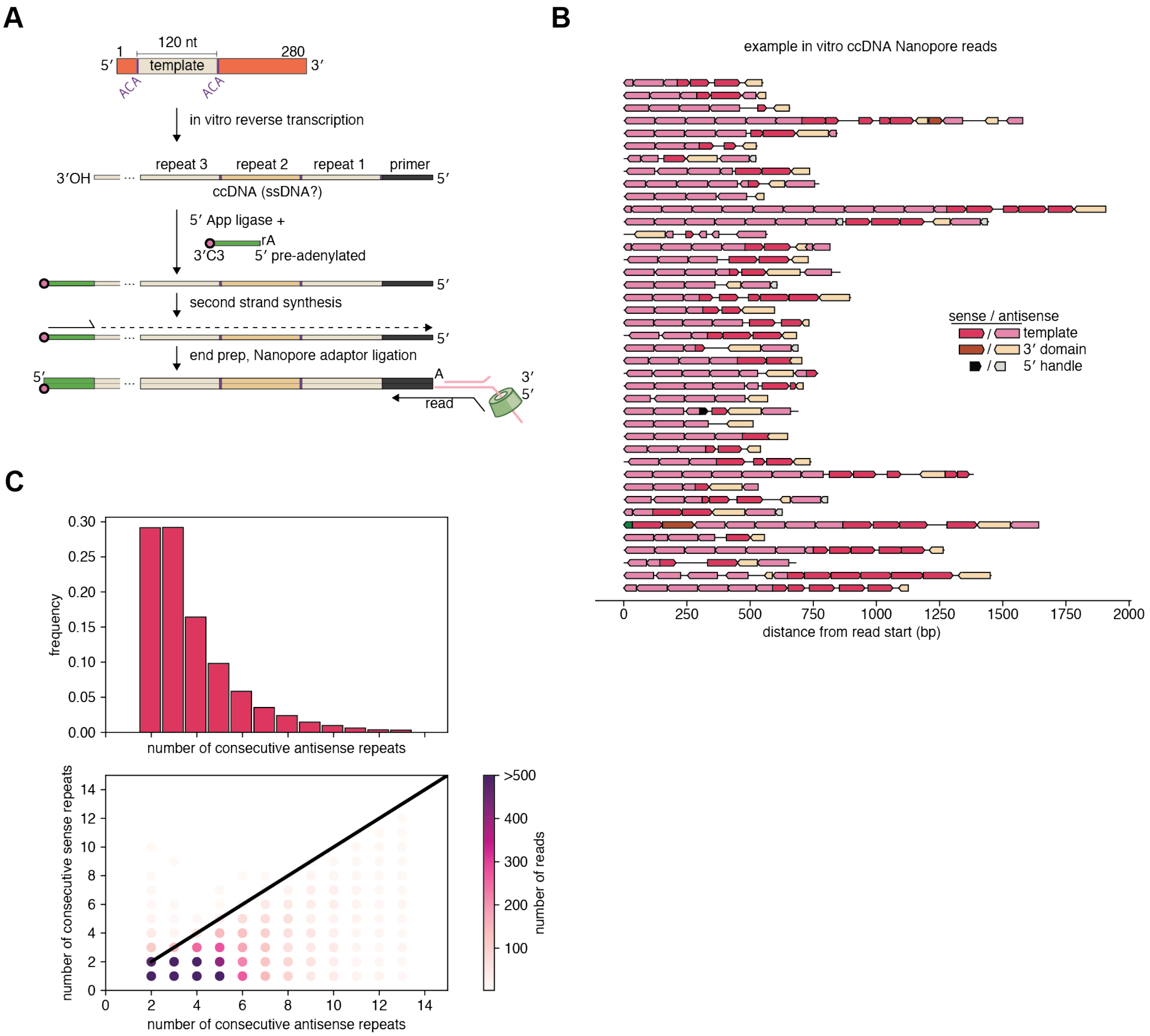


**Fig. S8.**

Nanopore sequencing of in vitro synthesized ccDNA. **A)** Schematic of Nanopore library preparation method. The method was designed to allow sequencing of blunt-ended dsDNA present, which would naturally be ligated to the Nanopore adaptor during the final step, as well as ssDNA or dsDNA with ssDNA overhangs. **B)** Randomly-selected example Nanopore reads, with the indicated features annotated using RepeatMasker. **C)** Distributions of the number of antisense and sense repeats in each Nanopore read. The number of consecutive sense repeats is rarely more than the number of consecutive antisense repeats (above the y=x diagonal), supporting a model where the antisense repeats template synthesis of sense repeats.

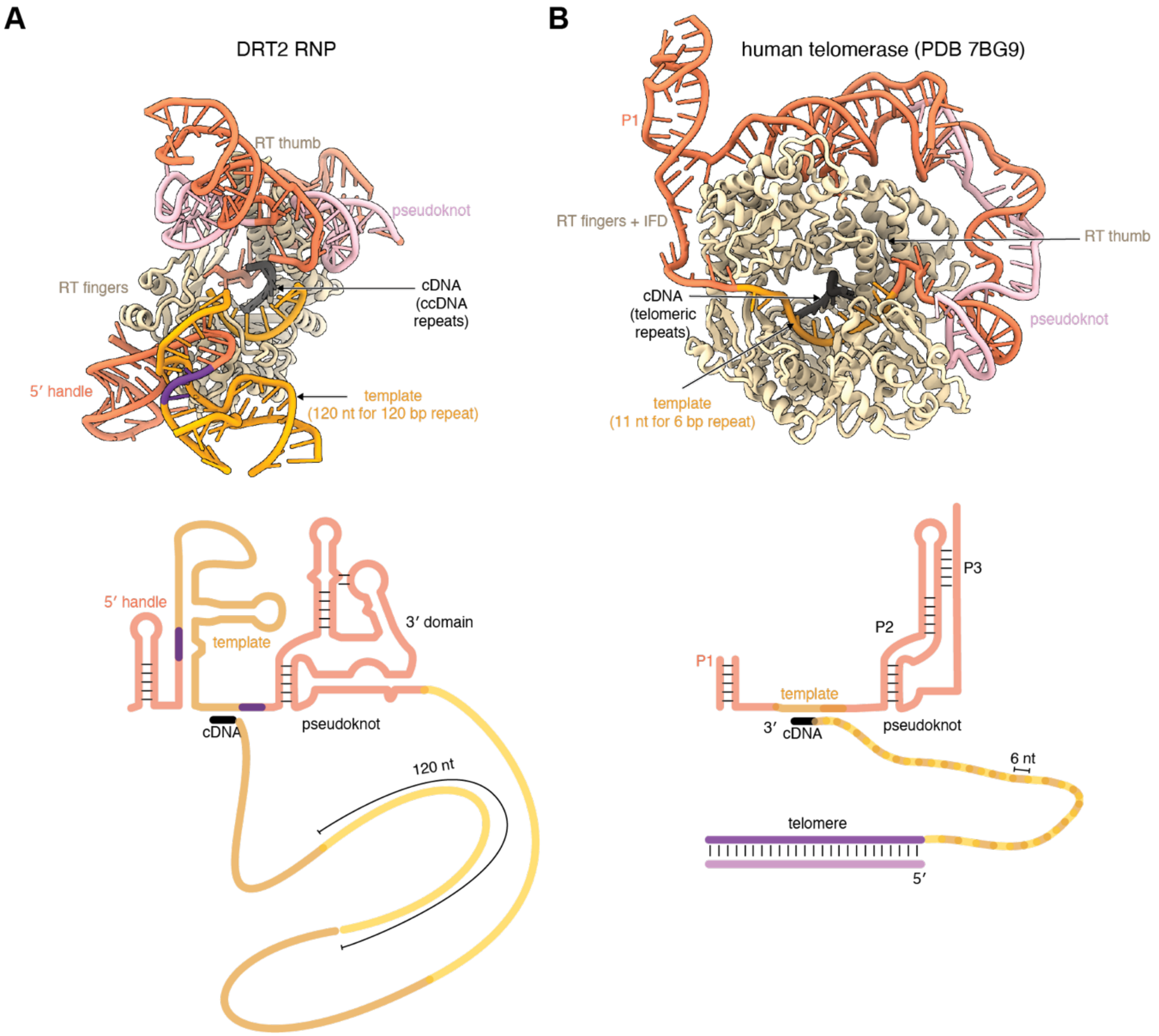


**Fig. S9.**

Comparison of DRT2 to telomerase. **A)** Structure of the DRT2 RNP and simplified schematic of its ncRNA component and repeat synthesis. **B)** The same view of the structure of human telomerase bound to telomeric DNA (*31*). Most of the telomerase RNA is hidden for clarity.

**Table S1.**

Oligonucleotide and plasmid sequences used in this study
<attached Excel spreadsheet>

**Table S2.**

Cryo-EM data collection, refinement, and validation statistics

| | #1 DRT2 RNP, resting (PDB 9C0I) (EMDB 45085) | #2 DRT2 RNP, elongating (PDB 9C0J) (EMDB 45086 |
|---|---|---|
| **Data collection and Processing** | | |
| Microscope | Thermo Scientific Titan Krios cryo TEM | |
| Voltage (keV) | 300 | |
| Camera | Gatan K3 | |
| Magnification | 130000 | |
| Pixel size at detector (Å/pixel) | 0.663 | |
| Total electron exposure ($e^-$/Å$^2$) | 40.7 | 40.3 |
| Exposure rate (e-/pixel/sec) | 19.9 | 20.4 |
| Number of frames collected during exposure | 40 | 40 |
| Defocus range (µm) | -0.9 to -2.3 | -1 to -2.7 |
| Automation software | EPU | |
| Energy filter slit width | 20 eV | |
| Micrographs collected (no.) | 28211 over 2 datasets | 18453 |
| Total extracted particles (no.) | 4628424 | 3313207 |
| Refined final particles (no.) | 230001 | 136601 |
| Point-group | C1 | C1 |
| Estimated error of translations/rotations | 0.407 Å / 1.058º | 0.702 Å / 2.086º |
| Resolution (global, Å) | | |
| FSC 0.5 (unmasked/masked) | 3.79 / 3.21 | 3.86 / 3.37 |
| FSC 0.143 (unmasked/masked) | 3.26 / 2.91 | 3.37 / 3.17 |
| Resolution range (local, Å) | 2.86 – 4.70 | 3.11 – 6.07 |
| Map sharpening *B* factor (Å$^2$) | -88.6 | -111.3 |
| Map sharpening method | relion_postprocess | relion_postprocess |
| 3D FSC sphericity | 0.980 | 0.972 |
| **Model composition** | | |
| Atoms (non-hydrogen) | 7701 | 7055 |
| Protein residues | 425 | 425 |
| Ligands | 2 | 3 |
| RNA/DNA bases | 197 | 165 |
| **Model Refinement** | | |
| Refinement package | phenix.real_space_refine | phenix.real_space_refine |
| - real or reciprocal space | Real space | Real space |
| - resolution cutoff | 2.9 | 3.1 |
| Model-Map scores | | |
| -CC | 0.88 | 0.87 |
| - Map-model FSC=0.5 | 2.9 Å | 3.2 Å |
| *B* factors (Å$^2$) | | |
| Protein residues | 27.1 | 18.0 |
| Ligands | 39.5 | 33.5 |
| RNA/DNA | 84.2 | 63.1 |
| R.m.s. deviations from ideal values | | |
| Bond lengths (Å) | 0.005 | 0.005 |
| Bond angles (°) | 0.953 | 1.007 |
| **Validation** | | |
| MolProbity score | 0.77 | 0.73 |
| CaBLAM outliers (%) | 0.71 | 0.71 |
| Clashscore | 0.89 | 0.72 |

| | | |
|---|---|---|
| Poor rotamers (%) | 0 | 0.52 |
| C-beta deviations (%) | 0 | 0 |
| EMRinger score | 5.17 | 5.16 |
| Ramachandran plot | | |
| Favored (%) | 98.8 | 98.8 |
| Outliers (%) | 0 | 0 |
